# Value-Gradient Generalist Design of Muscle–Tendon Parameters for Cross-Terrain Musculoskeletal Locomotion

**DOI:** 10.3390/biomimetics11090623

**Published:** 2026-09-02

**Authors:** Lidong Sun, Ye Wang, Hao Cha, Fuchun Sun

**Affiliations:** 1 Naval University of Engineering, Wuhan 430033, China; 0909041006@nue.edu.cn; 2 Department of Mechanical Engineering, Tsinghua University, Beijing 100084, China; wang-ye22@mails.tsinghua.edu.cn; 3 Department of Computer Science and Technology, Tsinghua University, Beijing 100084, China

**Keywords:** musculoskeletal locomotion, biomimetic robotics, Value-Gradient Generalist Design (VGGD), reinforcement learning, terrain-aware control

## Abstract

Compliant muscle–tendon mechanics can improve terrain adaptation in musculoskeletal robots, but heterogeneous terrains impose competing requirements on compliance, propulsion, foot clearance, and support transfer. This paper proposes Value-Gradient Generalist Design (VGGD), a framework for selecting one fixed physiological muscle–tendon parameterization for cross-terrain locomotion while preserving skeletal topology and muscle routing. VGGD searches a compact PCA-based manifold that coordinates bounded scale factors for muscle strength, contraction-velocity capacity, and passive elastic response. A design- and terrain-conditioned value proxy is learned from sampled latent designs and then optimized by proximity-regularized projected value-gradient ascent under a soft-worst objective. Independent proxy validation uses 50 random designs solely for calibration and 100 separately sampled test designs excluded from fitting and Stage-B optimization. On the 100-design test set, the proxy shows positive agreement with mean cross-terrain return (Pearson r=0.580, Spearman ρ=0.565) and worst-terrain return (r=0.583, ρ=0.551), with all bootstrap intervals above zero and Holm-adjusted permutation p=0.0006. A separate paired local-direction test uses 60 previously unused evaluation seeds and equal feasible-space perturbation radii; at the nominal, midpoint, and selected designs, the proxy-gradient direction agrees with improvements in mean and seed-wise worst-terrain rollout return. After design selection, the muscle–tendon parameters are fixed, and a terrain-aware controller is trained with variational information-bottleneck regularization and auxiliary expert distillation. Checkpoint-resolved evaluation records identify 216/300 successes and an overall mean distance of 12.14 m for the complete pipeline, compared with 57/300 and 6.20 m for nominal-body PPO. The three checkpoint success rates are 84%, 78%, and 54% for Proposed and 49%, 0%, and 8% for PPO; exact two-sided policy-level permutation tests yield p=0.10 for success rate and p=0.20 for mean distance. All methods receive the same nominal 100-million-step final-controller budget per training run, while the 10 M Stage-A budget and expert-pretraining costs are reported separately.

## 1. Introduction

Biological locomotion emerges from the interaction between neural commands and a compliant musculoskeletal body. Musculoskeletal robots embody this principle by generating motion through redundant muscle excitation, nonlinear muscle–tendon dynamics, compliant transmission, tendon elasticity, and passive body mechanics [1,2,3,4]. These properties allow the body to participate directly in impact absorption, elastic energy exchange, distributed actuation, and contact adaptation. They also make locomotion learning harder because performance depends not only on the control policy but also on the physiological parameterization of the muscle–tendon system.

Recent studies have improved simulated musculoskeletal control through reinforcement learning and muscle-synergy representations [4,5,6]. Related imitation-learning and morphology–controller co-optimization approaches provide complementary mechanisms for acquiring locomotor skills and adapting embodiment [7,8,9,10]. These methods have produced promising results in single-environment or task-specific settings. However, most learning-based frameworks either keep physiological muscle–tendon parameters fixed during policy optimization or adapt them jointly with the controller for a specific task or terrain. This coupling can bind learned body parameters to terrain-specific rewards, contact patterns, and control strategies. Consequently, a parameter configuration that is effective for flat walking may not provide sufficient compliance for rough contact, sustained propulsion for hilly terrain, or foot clearance and support transfer for stair traversal.

This limitation becomes more pronounced when the same musculoskeletal agent must traverse heterogeneous terrain. Optimizing separate muscle–tendon parameterizations for different terrains can improve task-specific performance, but it increases training cost, requires terrain-dependent body-parameter switching, and may fail when terrain conditions vary continuously. For biomimetic robots, terrain-dependent body-parameter switching is also undesirable: the physical body should remain fixed, while the controller adapts to changing environments.

**Figure 1:**
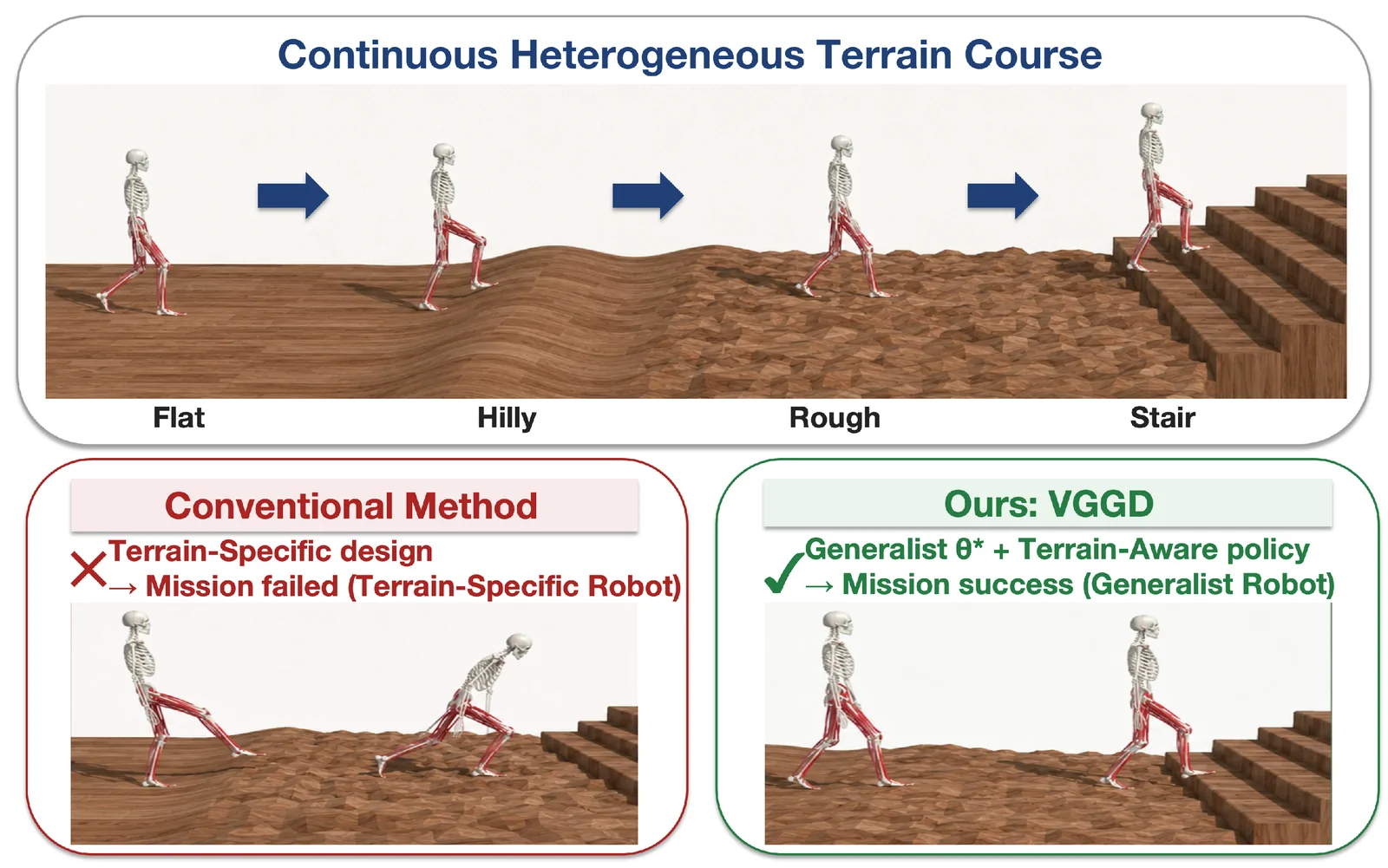
Motivation and evaluation setting of the proposed VGGD framework. The upper panel shows a continuous heterogeneous terrain course composed of flat, hilly, rough, and stair sections for evaluating cross-terrain locomotion under changing ground conditions. The lower panels compare terrain-specific physiological designs with the proposed generalist design. Terrain-specific physiological parameterizations may fail to transfer when terrain conditions change, whereas VGGD selects a single generalist muscle–tendon parameterization and trains a terrain-aware policy on the fixed body. The superscript asterisk denotes the selected design and its corresponding trained policy.

To address this problem, we propose Value-Gradient Generalist Design (VGGD), a value-guided framework that selects a fixed generalist muscle–tendon parameterization before training a terrain-aware controller on the selected body. In this work, morphology refers to the physiological muscle–tendon parameter configuration rather than to skeletal topology. VGGD first learns a terrain-conditioned value model from sampled parameter configurations, enabling candidate muscle–tendon designs to be scored under different terrain contexts. It then performs proximity-regularized projected value-gradient search in a compact PCA-based design manifold and decodes the selected latent design into coordinated bounded scalings of muscle strength, contraction-velocity capacity, and passive elastic response. The quadratic proximity term discourages large departures from the nominal design; it is not a classical trust-region method with an adaptive radius or acceptance test. After this design stage, the muscle–tendon parameters are fixed, and a unified terrain-aware controller is trained on the fixed body. This design–control separation avoids terrain-by-terrain body-parameter switching and provides an interpretable basis for testing whether one physiological parameterization can support locomotion across heterogeneous terrains. Figure 1 illustrates the motivation and evaluation setting. We construct a heterogeneous-terrain locomotion setting in MyoSuite that includes flat, rough, hilly, stair, and mixed terrain conditions. This setting enables us to evaluate whether a selected physiological muscle–tendon parameterization remains effective when the same musculoskeletal agent encounters different terrain-induced mechanical demands.

We evaluate the method at three complementary levels. First, held-out ranking and paired local-direction tests assess the design-search proxy. Second, traversal distance, completion rate, and learning curves characterize cross-terrain task performance. Third, gait-level analyses examine spatiotemporal variables, contact timing, joint trajectories, and muscle-activation structure.

The main contributions of this paper are summarized as follows:

1. We introduce a heterogeneous-terrain musculoskeletal locomotion setting in MyoSuite and formulate it as a generalist physiological muscle–tendon parameter-design problem. Rather than optimizing separate parameter configurations for different terrains, the design stage identifies one fixed physiological configuration over the flat, rough, hilly, and stair tasks; the same fixed body is then additionally evaluated on a mixed course that comprises the primitive terrain sections.
2. We propose VGGD, a value-guided design framework that learns a terrain-conditioned value model and applies proximity-regularized projected value-gradient search in a compact PCA-based physiological manifold. The selected design is fixed before final controller training, decoupling generalist body-parameter selection from subsequent terrain-aware policy optimization.
3. We independently validate the frozen design proxy on a separate calibration set and 100 genuinely held-out random designs, reporting design-level correlations, uncertainty intervals, terrain-wise errors, calibration, and local ranking evidence. We also report checkpoint-resolved task outcomes with exact observed success counts and policy-level uncertainty summaries, and separate the original illustrative cross-method gait traces from a Proposed-only multi-seed gait-repeatability analysis.

## 2. Related Work

### 2.1. Biomechanics and Musculoskeletal Robot Learning

Biological locomotion is commonly interpreted as the product of coupled neural control, muscle–tendon mechanics, limb dynamics, and environmental contact, rather than as a sequence of independent joint commands [11,12,13,14]. This view is especially relevant for uneven-terrain locomotion, where intrinsic limb compliance, support transfer, and terrain-dependent foot placement contribute to stability and energetic economy [15,16,17,18]. Classical gait analysis and biomechanical modeling further show that locomotor performance is shaped by spatiotemporal gait variables, joint coordination, muscle activation patterns, and body-size scaling [19,20,21,22,23]. These studies motivate biomimetic robots whose actuation and control are mediated by compliant muscle–tendon structures rather than by direct torque sources alone.

Muscle-driven simulation builds on Hill-type muscle modeling, in which active force–length behavior, force–velocity behavior, activation dynamics, tendon compliance, and passive elasticity jointly determine force generation [2,3,24,25]. The functional roles of these components have been extensively studied in human locomotion. For example, plantar-flexor force production contributes to support, forward progression, and swing initiation, while muscle forces redistribute segmental power during walking [26,27,28]. Springs and compliant elements also contribute to impact absorption, elastic energy exchange, and passive stabilization in legged locomotion and embodied intelligence [1,29,30]. These findings support the central premise of this work: muscle–tendon parameters should be treated as part of the functional locomotor substrate, not merely as fixed simulator constants.

Open-source physics and musculoskeletal platforms have made it possible to train and evaluate high-dimensional muscle-driven agents at scale. OpenSim provides a widely used platform for musculoskeletal dynamics and neuromuscular simulation [31,32]; MuJoCo provides efficient contact-rich dynamics for model-based and reinforcement-learning control [33]; and MyoSuite provides contact-rich musculoskeletal tasks with physiologically motivated actuation models [4]. On these platforms, reinforcement-learning and representation-learning methods have improved the control of overactuated musculoskeletal systems. DEP-RL improves exploration through action-correlated embodied noise [34]; hierarchical and synergy-based approaches reduce the effective control dimension [5,35,36,37,38,39]; DynSyn extracts dynamical synergistic representations from system structure [6]; and Diff-Muscle uses a structured mapping from joint-space planning to muscle activation for dexterous musculoskeletal control [40]. These methods primarily improve controller learning. In contrast, VGGD focuses on whether a fixed but deliberately selected physiological muscle–tendon parameterization can support locomotion across heterogeneous terrains.

### 2.2. Muscle-Parameter Design and Morphology Optimization

Morphology–control co-design studies how physical parameters and policies can be optimized jointly. Early evolutionary robotics demonstrated that morphology and behavior can co-evolve for task adaptation [41,42]. Later studies emphasized morphological computation and learning-based embodiment design, showing that body structure, perception, action, and control are tightly coupled in embodied agents [43,44,45]. Reinforcement-learning-based co-design methods further optimized body attributes and policies together, including data-efficient co-adaptation [8], deep reinforcement-learning-based design–control optimization [46], and morphology optimization for embodied agents [47]. Transform2Act treats design transformations as part of the decision process [9]; SARD exploits structured symmetry in robot design spaces [10]; CompetEvo studies morphology evolution in competitive scenarios [48]; and BodyGen improves embodiment co-design through topology-aware representations and temporal credit assignment [49].

Although these studies demonstrate the value of embodiment adaptation, most focus on skeletal topology, link geometry, joint attributes, or generic morphology. Muscle-driven systems require a more restricted and physiologically interpretable design view. In musculoskeletal models, relevant design variables include peak isometric force, contraction-velocity capacity, passive elastic response, tendon compliance, and related model-level parameters [2,3,25]. Recent musculoskeletal co-design studies have begun to tune such parameters directly. Bayesian morphology optimization showed that muscle parameters can be optimized for musculoskeletal systems [50], and spectral or PCA-based design methods construct low-dimensional physiological parameter spaces to avoid independent per-muscle perturbations [51]. Our work follows this physiological parameter-design perspective but makes a different optimization commitment: instead of finding a terrain-specific morphology or co-adapting the body throughout final policy training, VGGD selects one reusable muscle–tendon parameterization and fixes it before terrain-aware controller learning.

### 2.3. Terrain-Aware and Generalist Locomotion Learning

Terrain-aware locomotion learning aims to preserve locomotor performance when ground geometry and contact conditions change. Legged-robot studies commonly use terrain height maps, privileged observations, latent terrain representations, recurrent histories, or teacher–student adaptation to improve robustness over rough terrain, slopes, stairs, and outdoor environments [52,53,54]. Related physics-based character-control methods show that imitation and adversarial motion priors can produce coordinated whole-body skills, but they typically assume a fixed body and focus on policy learning rather than physiological parameter selection [7,55]. These studies establish the importance of terrain context for control; they do not directly answer whether one fixed muscle–tendon design can serve as a reusable mechanical substrate across terrains.

A related question is how much terrain information should be exposed to the final controller. High-dimensional exteroceptive observations can improve terrain specificity, but they may also increase sensitivity to nuisance details. Information-bottleneck theory provides a principled way to retain task-relevant information while suppressing redundant variation [56,57]. In locomotion, compact terrain embeddings can help the policy respond to contact-relevant terrain features without making the body design itself terrain-dependent. Knowledge distillation offers another route to generalist control by transferring information from specialist or privileged policies to a deployable student policy [58,59]. In VGGD, these terrain-aware control mechanisms are used only after the physiological parameterization has been selected and fixed. This separation prevents expert information or terrain-specific policies from directly modifying the selected muscle–tendon parameters.

### 2.4. Position of This Work

The literature above leaves a gap between muscle-driven controller learning, embodiment co-design, and terrain-aware policy adaptation. Controller-learning methods usually assume fixed physiological parameters; morphology co-design methods often optimize task-specific bodies; and terrain-aware locomotion methods typically adapt the policy rather than the mechanical substrate. VGGD addresses this gap by formulating cross-terrain locomotion as a generalist muscle–tendon parameter-selection problem. The selected parameterization is constrained to a compact physiological design manifold, optimized through a terrain-conditioned value surrogate, and then fixed for final controller training. This formulation preserves the biomimetic interpretation of muscle–tendon properties while avoiding terrain-specific body-parameter switching.

## 3. Preliminaries and Problem Formulation

### 3.1. Physiological Muscle–Tendon Parameterization

In this paper, *morphology* has a restricted meaning: it refers to the simulator-level physiological muscle–tendon parameterization of a fixed musculoskeletal model, not to skeletal topology, link geometry, muscle routing, tendon attachment, or anatomical reconstruction. The anatomical structure, joint layout, and muscle routing are kept unchanged throughout the study. Only selected actuator-level physiological parameters are modified.

Following Hill-type muscle modeling and the SDE-style muscle–tendon property triad, we consider three implementation-level scale channels: peak force, contraction-velocity capacity, and passive-force response [2,3,24,25,51]. To make the ordering consistent with the block PCA operator below, the group-level design vector is channel-major:

(1) $$\theta ={\left[\sigma^{⊤},\nu^{⊤},\kappa^{⊤}\right]}^{⊤}\in D\subset R^{3M},\;\sigma ,\nu ,\kappa \in R^{M}.$$

Here $\sigma_{i}$ multiplies the nominal peak-force capacity, $\nu_{i}$ multiplies the nominal maximum contraction velocity, and $\kappa_{i}$ multiplies the nominal passive-force coefficient of muscle group *i*. The nominal design is $\bar{\theta }=1_{3M}$. The numerical mapping used in the experiments satisfies

(2) $$0.8<\sigma_{i}<1.5,\;0.5<\nu_{i}<1.5,\;0.8<\kappa_{i}<1.2,$$

with the asymmetric force range arising from the implementation in Equation (6). These are conservative simulator bounds around the nominal MyoSuite actuator; they are not claimed to be subject-specific physiological confidence intervals.

Let $\ell_{i}$ be actuator length, ${\dot{\ell }}_{i}$ actuator velocity, $\ell_{0,i}$ the optimal fiber length, and $\ell_{T,i}$ the tendon-length offset used by the MuJoCo muscle actuator. The normalized length and velocity supplied to the built-in muscle curves are

(3) $$L_{i}=\frac{\ell_{i}-\ell_{T,i}}{\ell_{0,i}},\;{\hat{V}}_{i}\left(\nu_{i}\right)=\frac{{\dot{\ell }}_{i}}{\ell_{0,i}\;\nu_{i}v_{\max,i}^{\mathrm{ref}}}.$$

Thus, ${\dot{\ell }}_{i}$ has units of length/time, whereas ${\hat{V}}_{i}$ is dimensionless; negative velocity denotes shortening and positive velocity denotes lengthening. The active force–length, active force–velocity, and passive force–length curve definitions remain those of the MuJoCo muscle actuator. At the level needed here, the scaled pulling force is summarized as

(4) $$f_{i}\left(\theta \right)=-F_{0,i}^{\mathrm{ref}}\left[\sigma_{i}a_{i}F_{L}\left(L_{i}\right)F_{V}\left({\hat{V}}_{i}\left(\nu_{i}\right)\right)+\kappa_{i}F_{P}\left(L_{i}\right)\right],$$

where the leading minus sign follows the actuator convention that muscle tension pulls along the tendon path. Equation (4) is a semantic summary of the three parameter channels; the simulator evaluates its native piecewise muscle curves rather than the exponential passive-force expression used in the original manuscript. Consequently, no removable singularity at a zero passive-shape parameter is introduced by VGGD.

### 3.2. Compact Physiological Design Manifold

Directly optimizing $\theta \in R^{3M}$ is difficult because the number of muscle groups can be large and independent per-muscle perturbations can generate fragmented, hard-to-interpret designs. To obtain a compact design space, VGGD uses a PCA-based physiological manifold inherited from spectral muscle-parameter co-design. In the SDE formulation, dynamic muscle–tendon length-response trajectories are collected under exploratory motion, bilateral muscle groups are tied by a symmetry prior, and PCA is applied to the grouped response covariance to obtain *K* dominant functional modes [51,60]. Let $W\in R^{M\times K}$ denote the resulting group-level basis and partition the latent variable as $z={\left[z_{\sigma }^{⊤},z_{\nu }^{⊤},z_{\kappa }^{⊤}\right]}^{⊤}\in {\left[-1,1\right]}^{3K}$. We expand the group-level basis consistently with the channel-major ordering in Equation (1):

(5) $$\tilde{W}=I_{3}⊗W\in R^{3M\times 3K},$$

where $I_{3}$ is the identity matrix over the (σ,ν,κ) channels and ⊗ denotes the Kronecker product. Define $q_{\sigma }=Wz_{\sigma }$, $q_{\nu }=Wz_{\nu }$, and $q_{\kappa }=Wz_{\kappa }$. The implementation decodes these coordinates through bounded nonlinear scale maps,

(6) $$\begin{array}{cc} \sigma_{i}\left(z\right) & =\left\{\begin{array}{cc} 1+0.5\tanh(q_{\sigma ,i}), & q_{\sigma ,i}\geq 0,\\ 1+0.2\tanh(q_{\sigma ,i}), & q_{\sigma ,i}<0, \end{array}\right.\\ \nu_{i}\left(z\right) & =1+0.5\tanh(q_{\nu ,i}),\\ \kappa_{i}\left(z\right) & =1+0.2\tanh(q_{\kappa ,i}), \end{array}$$

and therefore

(7) $$\theta =f_{\mathrm{morph}}\left(z\right)={\left[\sigma {\left(z\right)}^{⊤},\nu {\left(z\right)}^{⊤},\kappa {\left(z\right)}^{⊤}\right]}^{⊤}.$$

The group-level scales are broadcast to the paired left/right actuators belonging to each muscle group. At z=0, all multipliers equal one. This formulation does not change the anatomical skeleton or muscle routing; it restricts parameter search to coordinated group modes and makes the numerical ranges and monotonicity of every channel explicit.

The latent coordinates in Table 1 are the archived final Stage-B iterate associated with the completed Stage-A and buffer artifacts used for the revision audit. Several coordinates lie on the projection boundary. Section 5.3 therefore evaluates equal-distance feasible perturbations around the selected point; this local test does not turn the archived coordinates into physiological optima.

### 3.3. Terrain-Conditioned Locomotion MDP

Let $C_{\mathrm{design}}$ denote the four primitive terrain tasks used by Stages A–B, and let $C_{\mathrm{control}}=C_{\mathrm{eval}}$ denote the Stage-C training and reporting set,

(8) $$\begin{array}{cc} C_{\mathrm{design}} & =\{\mathrm{flat},\mathrm{rough},\mathrm{hilly},\mathrm{stair}\},\\ C_{\mathrm{control}}=C_{\mathrm{eval}} & =C_{\mathrm{design}}\cup \left\{\mathrm{mixed}\right\}. \end{array}$$

The mixed course composes sections from the primitive terrains. It is introduced after design selection and is used during Stage-C controller learning and evaluation. Each terrain $c\in C_{\mathrm{eval}}$ induces a terrain-conditioned MDP parameterized by the fixed physiological design θ,

(9) $$M_{\theta ,c}=\langle S,U,P_{\theta ,c},r_{c},\rho_{0,c},\gamma \rangle ,$$

where S is the continuous state space, $U\subseteq {\left[0,1\right]}^{n_{u}}$ is the muscle-excitation action space, $P_{\theta ,c}$ is the transition kernel, $r_{c}$ is the task reward, $\rho_{0,c}$ is the initial-state distribution, and γ∈[0,1) is the discount factor [61,62]. The dependence of $P_{\theta ,c}$ on both θ and *c* captures the fact that muscle–tendon parameters alter the body dynamics, while terrain geometry alters contact dynamics.

For a terrain-aware policy $\pi_{\phi }\left(a_{t}|o_{t},\eta_{c}\right)$, $o_{t}$ is the proprioceptive observation and $\eta_{c}$ is a compact terrain descriptor or learned terrain embedding. Let τ denote a rollout trajectory. Its probability density is

(10) $$p\left(\tau |\theta ,\phi ,c\right)=\rho_{0,c}\left(s_{0}\right)\prod_{t=0}^{T-1}\pi_{\phi }\left(a_{t}|o_{t},\eta_{c}\right)P_{\theta ,c}\left(s_{t+1}|s_{t},a_{t}\right).$$

The expected return on terrain *c* is then

(11) $$J_{c}\left(\theta ,\phi \right)=E_{\tau ∼p(\tau |\theta ,\phi ,c)}\left[\sum_{t=0}^{T-1}\gamma^{t}r_{c}\left(s_{t},a_{t}\right)\right].$$

This definition makes explicit that $J_{c}$ evaluates a policy on a fixed body under a specified terrain.

### 3.4. Generalist Muscle-Parameter Design Objective

The design-stage goal is to select one fixed generalist design over the primitive terrains $C_{\mathrm{design}}$. For any candidate design θ, define the terrain-aware controller that would ideally be obtained on that fixed body as

(12) $$\phi^{★}\left(\theta \right)=\arg\max_{\phi }\;E_{c∼p_{C_{\mathrm{design}}}}\left[J_{c}\left(\theta ,\phi \right)\right],$$

where $p_{C_{\mathrm{design}}}$ is the design-stage distribution over primitive terrains. A theoretically ideal generalist design can then be written as the bilevel objective

(13) $$\theta^{★}=\arg\max_{\theta \in f_{\mathrm{morph}}\left(Z\right)}A_{c\in C_{\mathrm{design}}}\left[J_{c}\left(\theta ,\phi^{★}\left(\theta \right)\right)\right],$$

where $A_{c\in C_{\mathrm{design}}}\left[\cdot \right]$ denotes a generic terrain-wise aggregation operator. In this work, we instantiate it as the soft-worst operator $A_{\tau }^{\mathrm{sw}}$ defined below.

(14) $$A_{\tau }^{\mathrm{sw}}\left({\left\{x_{c}\right\}}_{c\in C_{\mathrm{design}}}\right)=-\tau \log\left(\frac{1}{|C_{\mathrm{design}}|}\sum_{c\in C_{\mathrm{design}}}\exp\left(-\frac{x_{c}}{\tau }\right)\right),\;\tau >0.$$

which approaches $\min_{c\in C_{\mathrm{design}}}x_{c}$ as $\tau \to 0^{+}$. This choice discourages designs that perform well only on easy terrains.

Directly solving Equation (13) would require retraining a converged controller for every candidate physiological design. VGGD instead trains a shared design-conditioned Stage-A policy $\pi_{\phi }^{A}$ and transfers its frozen critic to Stage B for efficient latent-design scoring and gradient-based search.

After the selected latent design $z^{★}$ is decoded as $\theta^{★}=f_{\mathrm{morph}}\left(z^{★}\right)$, the physiological parameters are fixed and the final terrain-aware controller is trained over $C_{\mathrm{control}}$, including the mixed course, as 

(15) $$\phi^{★}=\arg\max_{\phi }\;E_{c∼p_{C_{\mathrm{control}}}}\left[J_{c}\left(\theta^{★},\phi \right)\right].$$

The design stage, therefore, selects one physiological muscle–tendon parameterization, and the control stage learns to exploit that fixed body across terrains. The mixed course is introduced only after design selection.

## 4. Value-Gradient Generalist Design

### 4.1. Framework Overview

**Figure 2:**
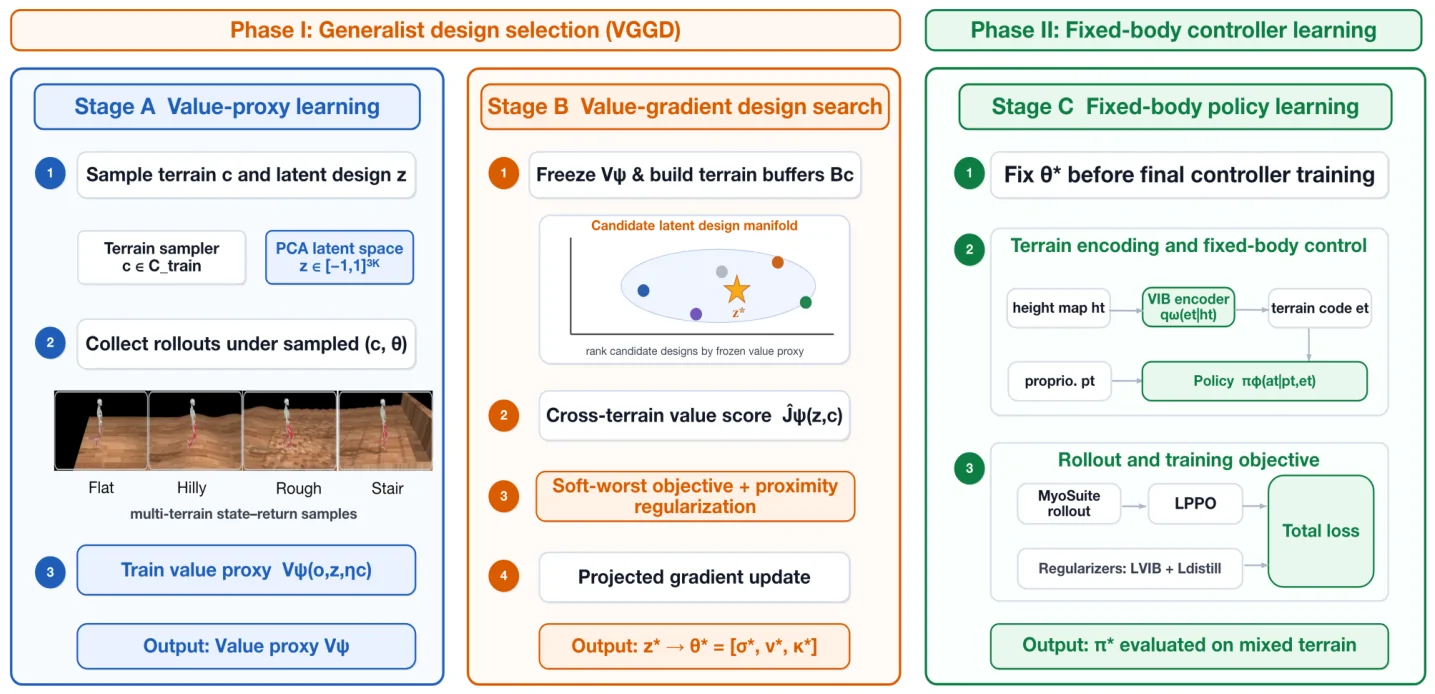
Overview of the proposed VGGD framework. Stage A samples latent physiological designs and terrains to learn a design- and terrain-conditioned value proxy. Stage B freezes this proxy and performs proximity-regularized projected value-gradient search to obtain the final iterate $z_{K}$. Stage C fixes the decoded muscle–tendon parameterization $\theta^{★}=f_{\mathrm{morph}}\left(z_{K}\right)$ and trains a terrain-aware controller on the fixed body. Blue, orange, and green denote Stages A, B, and C, respectively; numbered circles indicate the processing order.

VGGD approximates the bilevel generalist-design objective in Equation (13) without retraining a controller for every candidate’s physiological design. As illustrated in Figure 2, it contains two phases and three stages. Phase I performs fixed-body design selection. Stage A learns a design- and terrain-conditioned value proxy over sampled latent designs. Stage B freezes this proxy and uses its gradients to search for one generalist latent design in the compact manifold Z. Phase II performs controller learning on the selected body. Stage C fixes $\theta^{★}$ and trains the terrain-aware locomotion policy with PPO, a variational information-bottleneck terrain encoder, and auxiliary expert distillation.

This separation is important for both interpretation and evaluation. The physiological muscle–tendon parameters are updated only before Stage C. Once $z^{★}$ is decoded as $\theta^{★}=f_{\mathrm{morph}}\left(z^{★}\right)$, the body is fixed and no terrain-dependent body-parameter switching is allowed. Thus, any terrain adaptation during deployment must come from the controller rather than from changing muscle–tendon parameters. The term *value-gradient* refers to differentiating the learned value proxy with respect to the latent design *z*; it does not require differentiating through the MuJoCo dynamics or contact model.

Algorithm 1 summarizes the complete three-stage procedure.
**Algorithm 1** Value-Gradient Generalist Design**Require:** 
Compact basis $\tilde{W}$, latent domain Z, design terrains $C_{\mathrm{design}}$, control terrains $C_{\mathrm{control}}$, reference latent design $z_{\mathrm{ref}}$**Ensure:** 
Fixed generalist design $\theta^{★}$ and final terrain-aware policy $\pi_{\varphi }$  1:Initialize Stage-A rollout policy $\pi_{\phi }^{A}$ and value proxy $V_{\psi }$  2:**for** Stage-A value-learning iterations **do**  3:     Sample terrain $c∼p_{C_{\mathrm{design}}}$ and latent design $z∼q_{Z}$  4:     Decode and apply $\theta =f_{\mathrm{morph}}\left(z\right)$ using Equation (7)  5:     Collect rollouts in $M_{\theta ,c}$ with augmented input $x_{t}=\left[o_{t},z,\eta_{c}\right]$  6:     Update $\pi_{\phi }^{A}$ and $V_{\psi }$ using PPO  7:**end for**  8:Freeze $V_{\psi }$ and construct terrain-wise observation buffers ${\left\{B_{c}\right\}}_{c\in C_{\mathrm{design}}}$  9:Initialize $z\leftarrow z_{\mathrm{ref}}$10:**for** Stage-B design-search iterations **do**11:     **for all** $c\in C_{\mathrm{design}}$ **do**12:          Estimate ${\hat{J}}_{\psi }\left(z,c\right)$ by averaging $V_{\psi }\left(\left[o,z,\eta_{c}\right]\right)$ over $B_{c}$13:     **end for**14:     Compute $L_{\mathrm{design}}\left(z\right)$ using soft-worst aggregation and quadratic proximity regularization15:     Update $z\leftarrow \Pi_{Z}\left(z+\alpha \nabla_{z}L_{\mathrm{design}}\left(z\right)\right)$16:**end for**17:Set $z^{★}\leftarrow z_{K}$ and $\theta^{★}=f_{\mathrm{morph}}\left(z_{K}\right)$18:Fix $\theta^{★}$19:**for** Stage-C controller-training iterations **do**20:     Encode local terrain observation $h_{t}$ into latent terrain code $e_{t}∼q_{\omega }\left(e_{t}|h_{t}\right)$21:     Train $\pi_{\varphi }\left(a_{t}|p_{t},e_{t}\right)$ with PPO, VIB regularization, and auxiliary expert distillation22:**end for**23:**return** $\theta^{★},\pi_{\varphi }$

### 4.2. Stage A: Design- and Terrain-Conditioned Value Learning

Stage A learns a differentiable surrogate that predicts the locomotion value of a candidate physiological design under a specified terrain context. At the beginning of each rollout, a terrain $c\in C_{\mathrm{design}}$ and a latent design z∈Z are sampled. The latent design is decoded into the full muscle–tendon parameter vector $\theta =f_{\mathrm{morph}}\left(z\right)$ and applied to the simulator before the episode starts. During the rollout, *z* remains fixed, so the sampled design acts as an episode-level body parameter rather than as a time-varying control action.

The Stage-A policy and critic receive the augmented input

(16) $$x_{t}=\left[o_{t},z,\eta_{c}\right],$$

where $o_{t}$ is the musculoskeletal observation and $\eta_{c}$ is a compact terrain descriptor available during value-proxy training. PPO is used to train a rollout policy and critic,

(17) $$\pi_{\phi }^{A}\left(a_{t}|x_{t}\right),\;V_{\psi }\left(x_{t}\right)\approx E\left[\sum_{k=t}^{T-1}\gamma^{k-t}r_{c}\left(s_{k},u_{k}\right)|x_{t},\pi_{\phi }^{A}\right].$$

The Stage-A policy $\pi_{\phi }^{A}$ is not the final deployed controller. Its purpose is to expose the value proxy to diverse terrain–design combinations. The critic $V_{\psi }$ is the component transferred to Stage B because its dependence on *z* provides a differentiable local estimate of how design changes affect predicted return.

### 4.3. Stage B: Value-Gradient Generalist Design Selection

After Stage A, the value proxy is frozen. For each training terrain, we collect a terrain-wise observation buffer from multiple latent designs,

(18) $$B_{c}={\left\{o_{i}^{c}\right\}}_{i=1}^{N_{c}},\;c\in C_{\mathrm{design}}.$$

For a candidate latent design *z*, its predicted score on terrain *c* is estimated by re-evaluating the frozen critic on the buffered states while replacing only the design input,

(19) $${\hat{J}}_{\psi }\left(z,c\right)=\frac{1}{N_{c}}\sum_{i=1}^{N_{c}}V_{\psi }\left(\left[o_{i}^{c},z,\eta_{c}\right]\right).$$

This substitution keeps the buffered state distribution fixed. Consequently, ${\hat{J}}_{\psi }\left(z,c\right)$ and its derivative are local properties of the learned proxy, not unbiased estimates of either the on-policy rollout return or its simulator gradient at design *z*. The buffer spans multiple designs to reduce, but not eliminate, distribution shift. The archived implementation aggregates the terrain-wise means directly; it does not apply an additional terrain-wise standardization in Stage B. The scores are aggregated with the soft-worst operator defined in Equation (14),

(20) $$O_{\mathrm{sw}}\left(z\right)=A_{\tau_{d}}^{\mathrm{sw}}\left({\left\{{\hat{J}}_{\psi }\left(z,c\right)\right\}}_{c\in C_{\mathrm{design}}}\right),$$

where $\tau_{d}$ is the design-stage temperature parameter. This soft-worst formulation encourages the optimizer to improve predicted performance on the lowest-scoring terrains, rather than simply increasing the average score across terrains. The complete design objective is

(21) $$L_{\mathrm{design}}\left(z\right)=O_{\mathrm{sw}}\left(z\right)-\frac{\lambda_{\mathrm{prox}}}{3K}{∥z-z_{\mathrm{ref}}∥}_{2}^{2},$$

where $\lambda_{\mathrm{prox}}$ controls a quadratic proximity penalty and $z_{\mathrm{ref}}=0$ denotes the nominal design. We use the term *proximity regularization* rather than *trust region*: the implementation does not estimate a trust-region radius or accept/reject steps using actual improvement.

Because $V_{\psi }$ is differentiable with respect to *z*, the objective in Equation (21) can be optimized by projected gradient ascent. The gradient of the soft-worst term has the form

(22) $$\nabla_{z}O_{\mathrm{sw}}\left(z\right)=\sum_{c\in C_{\mathrm{design}}}w_{c}\left(z\right)\nabla_{z}{\hat{J}}_{\psi }\left(z,c\right),\;w_{c}\left(z\right)=\frac{\exp\left(-{\hat{J}}_{\psi }\left(z,c\right)/\tau_{d}\right)}{\sum_{c^{\prime }\in C_{\mathrm{design}}}\exp\left(-{\hat{J}}_{\psi }\left(z,c^{\prime }\right)/\tau_{d}\right)}.$$

Terrains with lower proxy scores receive larger weights, so the update preferentially targets the currently limiting proxy component. With an Adam step operator ${\mathrm{AdamStep}}_{\alpha }$, gradient clipping, and projection to $Z={\left[-1,1\right]}^{3K}$, the implemented update is summarized as

(23) $$z_{k+1}=\Pi_{Z}\left({\mathrm{AdamStep}}_{\alpha }\left[z_{k},\nabla_{z}L_{\mathrm{design}}\left(z_{k}\right)\right]\right),$$

where α is the design-search learning rate. The procedure performs $K_{B}=200$ updates and returns the final iterate; it does not claim to identify a global maximizer:

(24) $$z^{★}:=z_{K_{B}},\;\theta^{★}=f_{\mathrm{morph}}\left(z^{★}\right).$$

After this point, $\theta^{★}$ is fixed, and no further muscle–tendon parameter update is performed. Because the proxy gradient is evaluated on buffered states, the result should be interpreted as a proxy-guided candidate obtained within the prescribed manifold, not as proof of a locally optimal design under the true simulator return.

### 4.4. Stage C: Terrain-Aware Control on the Fixed Design

Stage C trains the final locomotion controller on the selected body. The controller receives a proprioceptive observation $p_{t}$ and a local terrain observation $h_{t}$, such as a height-map patch. A variational terrain encoder maps $h_{t}$ to a compact latent code,

(25) $$q_{\omega }\left(e_{t}|h_{t}\right)=N\left(\mu_{\omega }\left(h_{t}\right),\mathrm{diag}\left(\sigma_{\omega }^{2}\left(h_{t}\right)\right)\right),\;e_{t}∼q_{\omega }\left(e_{t}|h_{t}\right),$$

and the final policy is

(26) $$\pi_{\varphi }\left(a_{t}|p_{t},e_{t}\right).$$

The terrain encoder and policy are trained jointly with PPO to maximize Equation (15) on the fixed design $\theta^{★}$. During training, a variational information-bottleneck penalty encourages the terrain code to preserve task-relevant information while suppressing redundant terrain details,

(27) $$L_{\mathrm{VIB}}=E_{t}\left[{\left(D_{\mathrm{KL}}\left(q_{\omega }\left(e_{t}|h_{t}\right)\;∥\;N\left(0,I\right)\right)-\delta \right)}_{+}\right],$$

where ${\left(x\right)}_{+}=\max\left(x,0\right)$ and δ is a free-bits threshold. When δ=0, this reduces to the standard variational information-bottleneck regularizer. The VIB component is used only for controller learning; it does not participate in Stage-B design selection.

### 4.5. Auxiliary Terrain-Aware Expert Distillation

Pretrained terrain-specific expert policies can be used as auxiliary teachers during Stage C. Let $\pi_{j}^{E}$ denote the expert associated with terrain class *j*, and let $E(c_{t})$ map the current terrain descriptor or segment label to the corresponding expert. We regularize the student by matching the mean action of the selected expert,

(28) $$L_{\mathrm{distill}}=E_{t}\left[{∥\mu_{\varphi }\left(p_{t},e_{t}\right)-\mu_{E(c_{t})}^{E}\left(p_{t}\right)∥}_{2}^{2}\right],$$

where $\mu_{\varphi }$ and $\mu_{E(c_{t})}^{E}$ are the mean actions of the student policy and the selected expert. For mixed terrain, $c_{t}$ can change within a rollout, so the teacher is selected according to the current terrain segment rather than the episode label.

The minimized Stage-C training loss is

(29) $$L_{C}=L_{\mathrm{PPO}}+\beta_{\mathrm{VIB}}L_{\mathrm{VIB}}+\beta_{D}L_{\mathrm{distill}},$$

where $L_{\mathrm{PPO}}$ denotes the usual PPO loss written in minimization form. Distillation is a Stage-C training regularizer on the fixed body and does not update $z^{★}$ or $\theta^{★}$.

## 5. Experiments

This section addresses three questions. First, can the frozen value proxy rank held-out designs and provide a useful local search direction? Second, how does the complete VGGD-based pipeline perform across terrains under the fixed final-controller training budget? Third, is its task-level behavior accompanied by repeatable terrain-conditioned gait organization? The evaluation proceeds from implementation settings and proxy validation to system-level comparisons, component controls, and biomechanical analysis.

### 5.1. Experimental Setup

The experiments are conducted on the MyoSuite MyoLeg model [4] with 80 muscle excitations. The primitive evaluation terrains are flat, rough, hilly, and stair. The mixed terrain concatenates primitive sections into a longer heterogeneous course with an approximate total length of 26 m, and the order of terrain sections is randomized across mixed-terrain test rollouts. To retain the task definition used for the originally reported pass rates, the main comparison defines success as reaching the 13 m evaluation target on every terrain, including mixed terrain; 26 m full-course mixed rollouts are analyzed separately for gait robustness and are not substituted into the main pass table. Unless otherwise stated, all evaluation results use the VecNormalize statistics paired with each checkpoint and stochastic policy execution with deterministic=False. The main comparison uses three independently launched policy-training runs per method and the fixed evaluation-seed set 1000–1019 for each checkpoint and terrain. Each evaluation seed controls stochastic action sampling and terrain randomization, including mixed-course segment ordering. The trained policy is the inferential unit. We report each checkpoint’s observed success count, between-checkpoint mean and SD, a checkpoint-bootstrap interval, and an exact two-sided permutation test over the three-versus-three policy summaries. Table 2 summarizes the implementation and evaluation settings.

Table 3 separates simulator interactions from proxy-only computation. Stage A follows the confirmed 10 M environment-step budget; completed Stage-C and expert runs use the archived SB3 num_timesteps values, including rollout-block rounding. Evaluation rollouts and the diagnostic proxy check are excluded from training totals. The full-cost total assigns all four teacher-training runs to Proposed; the reusable-teacher total reports the cost when those pretrained teachers are treated as a shared artifact. W&B logs retain wall-clock time and throughput for the main Stage-C runs, but Proposed and the no-terrain baselines were trained on different GPU hosts. Two Proposed logs continued beyond the nominal checkpoint used for the 100 M-step evaluation, so their displayed wall times cover the retained full logs (up to 118.807 M steps), whereas the interaction total used for the matched Stage-C comparison remains the evaluated 100.008 M checkpoint per run. We therefore report the recorded ranges for auditability without treating them as a controlled GPU-hour comparison.

Accordingly, the performance comparison standardizes final-controller training at a nominal 100 M interactions per run. Table 3 reports the Stage-A, Stage-B, expert-pretraining, and Stage-C records separately so that the role and cost of each stage are explicit.

### 5.2. Independent Held-Out Value-Proxy Validation

Because Stage B uses a learned proxy rather than direct rollout optimization, we evaluate the frozen 10 M-step Stage-A proxy without additional policy training. Before the final evaluation, we fixed three disjoint candidate sets: 50 uniformly sampled latent designs used only to fit an affine mapping from proxy score to rollout return, 100 separately sampled random designs reserved for primary testing, and 32 feasible local designs used only for the neighborhood analysis in Section 5.3. The nominal and VGGD-selected designs are appended as descriptive anchors and excluded from calibration and all primary inferential statistics. The same complete 11,750-state cross-terrain buffer is used to score every candidate. Each design is evaluated with five common stochastic rollout seeds on each primitive terrain, and the design-level terrain mean—not an individual rollout—is the statistical unit.

The held-out correlations are positive for the aggregate objectives and all four terrains, with every confidence interval above zero. Kendall coefficients are 0.392 and 0.375 for mean and worst-terrain return, respectively. After calibration on the separate 50-design set, the test-set calibration slopes are 1.021 for mean return and 1.033 for worst-terrain return, and the corresponding mean observed-minus-predicted errors are −0.393 and 0.042. The 10-bin calibration curves and all candidate-level records are provided as Supplementary Figures S1 and S2 and machine-readable files. A separate terrain-identity diagnostic gives 39% agreement (κ=0.006); accordingly, we use the proxy to rank worst-terrain performance rather than to infer the categorical identity of the limiting terrain.

The descriptive anchors are reported separately, as requested. The nominal design has proxy score −1.656, mean return 78.617, and worst-terrain return 62.621; the VGGD-selected design has proxy score −1.292, mean return 72.679, and worst-terrain return 63.185. These anchor values are based on the same five common seeds per terrain but are not included in the 100-design correlations, confidence intervals, calibration fit, or significance tests. Thus, the primary conclusion from Table 4 is design-distribution ranking evidence for the frozen proxy, while the two named designs remain additional cases rather than validation samples.

### 5.3. Paired Local Proxy-Gradient Direction Validation

The ranking diagnostic above does not determine whether a proxy gradient computed on buffered states predicts the direction of a local change in simulator return. We therefore perform a paired direction test at three anchors: the nominal design $z_{\mathrm{nom}}=0$, the midpoint $z_{\mathrm{mid}}=0.5z^{★}$, and the selected design $z^{★}$. At an anchor *z*, let

(30) $$\hat{g}\left(z\right)=\frac{\nabla_{z}O_{\mathrm{sw}}\left(z\right)}{∥\nabla_{z}O_{\mathrm{sw}}{\left(z\right)∥}_{2}}$$

denote the unit proxy-gradient direction computed on the frozen cross-terrain buffer. The positive and negative candidates are

(31) $$z^{+}=\Pi_{Z}\;\left(z+\alpha_{+}\hat{g}\left(z\right)\right),\;z^{-}=\Pi_{Z}\;\left(z-\alpha_{-}\hat{g}\left(z\right)\right),$$

where $\alpha_{+}$ and $\alpha_{-}$ are solved separately so that

(32) $$∥z^{+}{-z∥}_{2}={∥z^{-}-z∥}_{2}=0.05.$$

Equal feasible-space radii are important because several components of $z^{★}$ lie on the box boundary. At $z^{★}$, the required ray scales are $\alpha_{+}=0.073601$ and $\alpha_{-}=0.050088$, while the actual projected distances are both 0.05.

To avoid reporting only a favorable perturbation scale, we first performed an exploratory sensitivity sweep over nominal ray scales {0.01,0.02,0.03,0.04,0.05,0.075,0.10} using evaluation seeds 1000–1019. The final equal-distance test then used a disjoint set of 60 previously unused seeds, 3000–3059. For each seed, $z^{-}$, *z*, and $z^{+}$ were evaluated with common random numbers on flat, rough, hilly, and stair terrains using the fixed Stage-A policy and the archived Stage-B inference path. The local test is an exception to the checkpoint-paired normalization statement in the general evaluation protocol: it uses the exact archived 10 M Stage-A checkpoint, cross-terrain buffer, and Stage-B/diagnostic inference path, and it does not substitute the separately saved later normalization statistics. Each anchor therefore contains 3×4×60=720 rollouts. We compute, within each seed, the mean return across terrains and the minimum return across terrains. Uncertainty is obtained by 10,000 paired bootstrap resamples at the seed level. Two-sided paired Wilcoxon signed-rank tests are reported as a secondary check.

Table 5 shows that the proxy score and all three rollout contrasts are positive at every anchor, with all paired-bootstrap intervals excluding zero. The terrain-specific positive-minus-negative differences are also positive, with intervals excluding zero on all four terrains. At the selected design, these differences are 0.898 [0.566, 1.296] on flat terrain, 0.960 [0.621, 1.354] on rough terrain, 1.409 [1.008, 1.937] on hilly terrain, and 0.868 [0.602, 1.133] on stair terrain. Across the three anchors and three aggregate rollout contrasts, all nine two-sided paired Wilcoxon tests remain significant after a conservative Bonferroni correction ($p_{\mathrm{adj}}<1.3\times 10^{-7}$).

We additionally evaluate 32 feasible designs at an equal $\ell_{2}$ radius of 0.05 from the selected design, disjoint from both random-design sets. For mean return, local ranking remains positive (Pearson r=0.563 [0.269, 0.765], permutation p=0.0009; Spearman ρ=0.481 [0.109, 0.739], p=0.0058). For worst-terrain return, the local coefficients are positive but less precise (r=0.277 [−0.100, 0.584], p=0.126; ρ=0.238 [−0.158, 0.569], p=0.192). The top-5 and top-10 overlaps are 2/5 and 4/10 for both targets. This neighborhood analysis complements, rather than replaces, the higher-powered paired gradient-ray experiment.

The combined evidence at the nominal, midpoint, and selected anchors shows that the frozen-buffer proxy supplies a rollout-consistent local gradient direction over the tested feasible-space neighborhood, while the random local cloud quantifies the finer-grained ranking uncertainty. The proxy has also been calibrated and tested independently in Section 5.2; nevertheless, its gradient is not the analytical derivative of simulator return, and the 60 seeds are evaluation repetitions for one fixed Stage-A policy rather than independently trained policies.

### 5.4. Compared Methods and Metrics

We evaluate VGGD using traveled distance, exact observed pass count, pass rate, training convergence, and gait-level descriptors. The archived per-rollout manifests provide pooled and checkpoint-specific success counts without rounding. The original cross-method gait traces are complemented by a Proposed-only robustness summary using two checkpoints and 20 common evaluation seeds per terrain.

The main comparison includes four deliberately constrained implementations. **Proposed** denotes the complete pipeline: proxy-guided selection of one fixed physiological design followed by terrain-aware controller learning with the VIB and distillation terms. **PPO** is a controller-only baseline on the nominal body and omits terrain height-map input. **T2A** is a fixed-output Transform2Act-style implementation without the VGGD objective. **SDE** uses the same compact PCA physiological manifold but not the VGGD soft-worst value-gradient selection objective. For T2A and SDE, the body is held fixed after their design stage to match the deployment constraint of one physical body. This restriction removes native online morphology adaptation and may disadvantage those algorithms relative to their original adaptive formulations. Accordingly, our results concern these fixed-body-constrained implementations and should not be generalized to unconstrained T2A or SDE.

Table 6 summarizes the components enabled in each evaluated configuration.

The component controls include **PPO + VIB + Distill**, which keeps the nominal body while matching the Stage-C controller components, and **VGGD + Distill**, which retains the selected body and distillation without VIB. We obtained one archived policy for each control and evaluated it with the same 20 stochastic seeds per terrain. The attempted VGGD+PPO artifact is not reported because its saved policy emitted a 95-dimensional action (80 muscle controls plus 15 design outputs), whereas the fixed-body Stage-C protocol requires an 80-dimensional muscle-control action.

For the reported final-controller runs, all methods use PPO, two 64-unit policy/value layers as instantiated in the archived SB3 checkpoints, the same nominal 100 M interaction budget, and the shared optimizer settings in Table 2; no method-specific hyperparameter sweep is used to favor Proposed. In the constrained T2A/SDE environments, the design component is applied at the initial design step and is then held fixed for the locomotion portion and evaluation. The baseline names in the tables, therefore, denote these modified fixed-output implementations, not exact reproductions of the original papers’ adaptive deployment protocols.

### 5.5. Task-Level Comparison with PPO, T2A, and SDE

**Figure 3:**
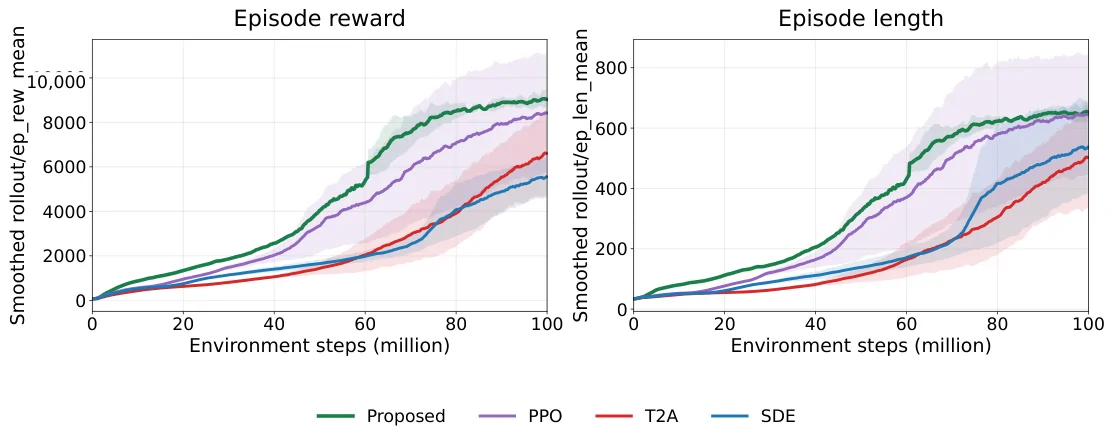
Training convergence of the main comparison group under the same 100 M-step final-controller budget. Curves report smoothed episode reward and episode length for Proposed, PPO, T2A and SDE. Shaded bands show the variability represented in the retained aggregate training records.

Figure 3 compares training trajectories under the same 100 M-step final-controller budget. Proposed and PPO reach high-return regimes earlier than several constrained design baselines. The curves characterize optimization behavior, while Table 7 reports the stochastic rollout outcomes.

#### Reporting Pass Outcomes and Uncertainty from Checkpoint-Resolved Records

The revision audit recovered the archived per-rollout manifests for all three main-comparison checkpoints of Proposed, PPO, T2A, and SDE. We therefore replace rounded mean counts with the actual observed pooled numerator and denominator and report each checkpoint separately in Table 8. The distance means and SDs in the original table are unchanged. The Wilson interval in Table 7 summarizes pooled rollout outcomes, while between-training variability is reported as the mean and SD of the three checkpoint summaries. A 10,000-resample checkpoint bootstrap provides an interval for the checkpoint mean, and two-sided exact permutation tests enumerate all 63=20 allocations of the three-versus-three policy summaries; the smallest attainable nonzero two-sided exact *p*-value is 0.10.

**Figure 4:**
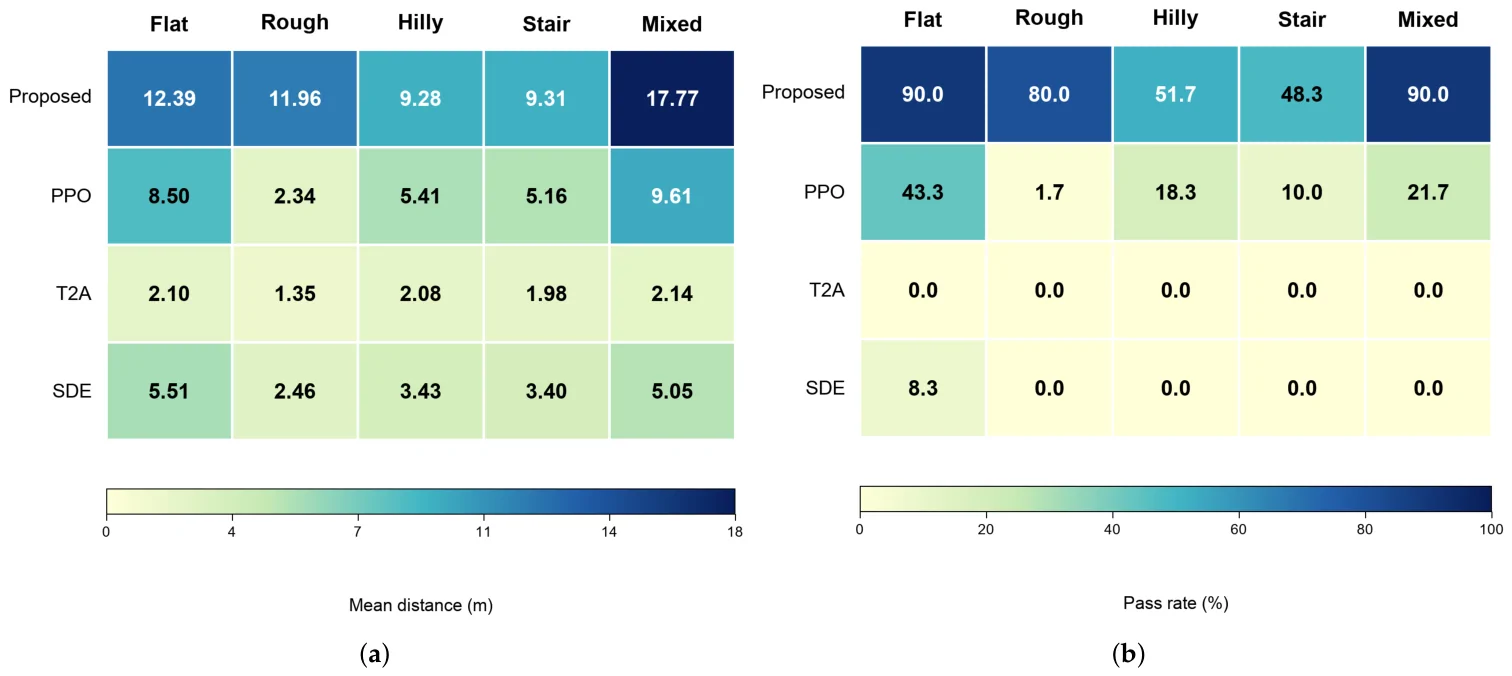
Terrain-wise visualization of the main comparison in Table 7. The distance panel preserves the original terrain-wise means, and the pass-rate panel uses the exact checkpoint-resolved pooled counts. (**a**) Mean distance, (**b**) Pass rate.

The complete Proposed pipeline retains the highest observed pass rate (216/300, 72.0%) and overall mean distance (12.14 m), compared with 57/300 (19.0%) and 6.20 m for nominal-body PPO. The corresponding pooled rollout-level Wilson intervals are [66.7, 76.8]% and [15.0, 23.8]%. T2A records 0/300 successes, and SDE records 5/300 (1.7%, which rounds to the originally printed 2%). The three Proposed checkpoints achieve 84, 78, and 54 successes out of 100, whereas the PPO checkpoints achieve 49, 0, and 8. At the policy level, the exact two-sided permutation tests yield p=0.10 for pass rate and p=0.20 for mean distance for Proposed versus PPO; neither test reaches the conventional 0.05 significance level. Figure 4 visualizes the terrain-wise means and exact pass rates reported in Table 7.

### 5.6. Ablation Study

The replicated Proposed result remains in Table 7, while Table 9 reports four component controls, each represented by one independently trained policy and 20 stochastic evaluation seeds per terrain. **PPO + VIB + Distill** keeps the nominal body and uses the terrain map, VIB, and expert-distillation mechanisms used by Proposed. **VGGD + Distill** removes VIB while retaining the selected body and distillation. The **SDE + VIB + Distill** and **SDE + VIB** variants use the PCA/SDE design formulation instead of VGGD. The attempted VGGD+PPO run is omitted because its 95-dimensional action space does not match the required 80-dimensional fixed-body Stage-C action protocol.

**Figure 5:**
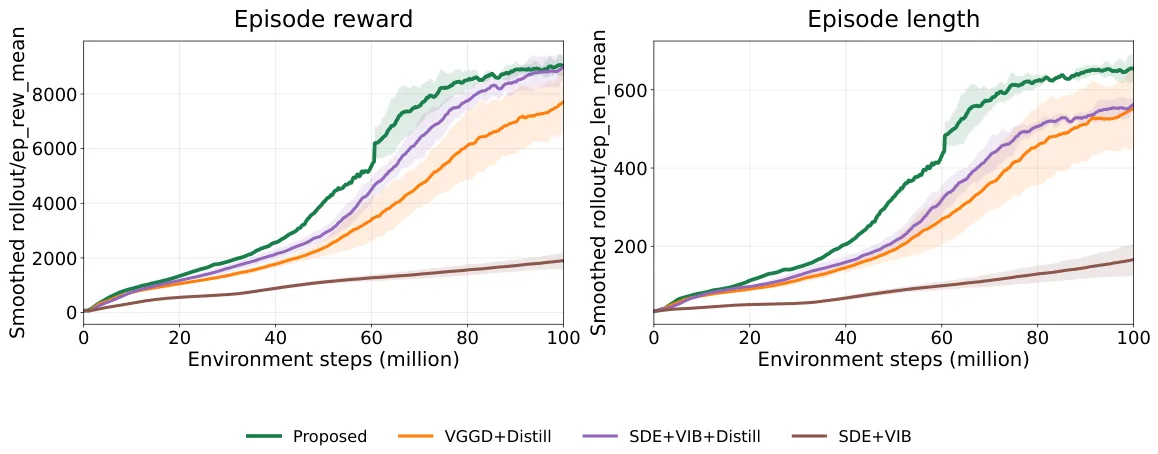
Archived ablation training curves under the nominal 100 M-step final-controller budget. Lines and shaded bands reproduce the retained aggregate plots. The added PPO + VIB + Distill rollout result is summarized in Table 9 and Figure 6.

**Figure 6:**
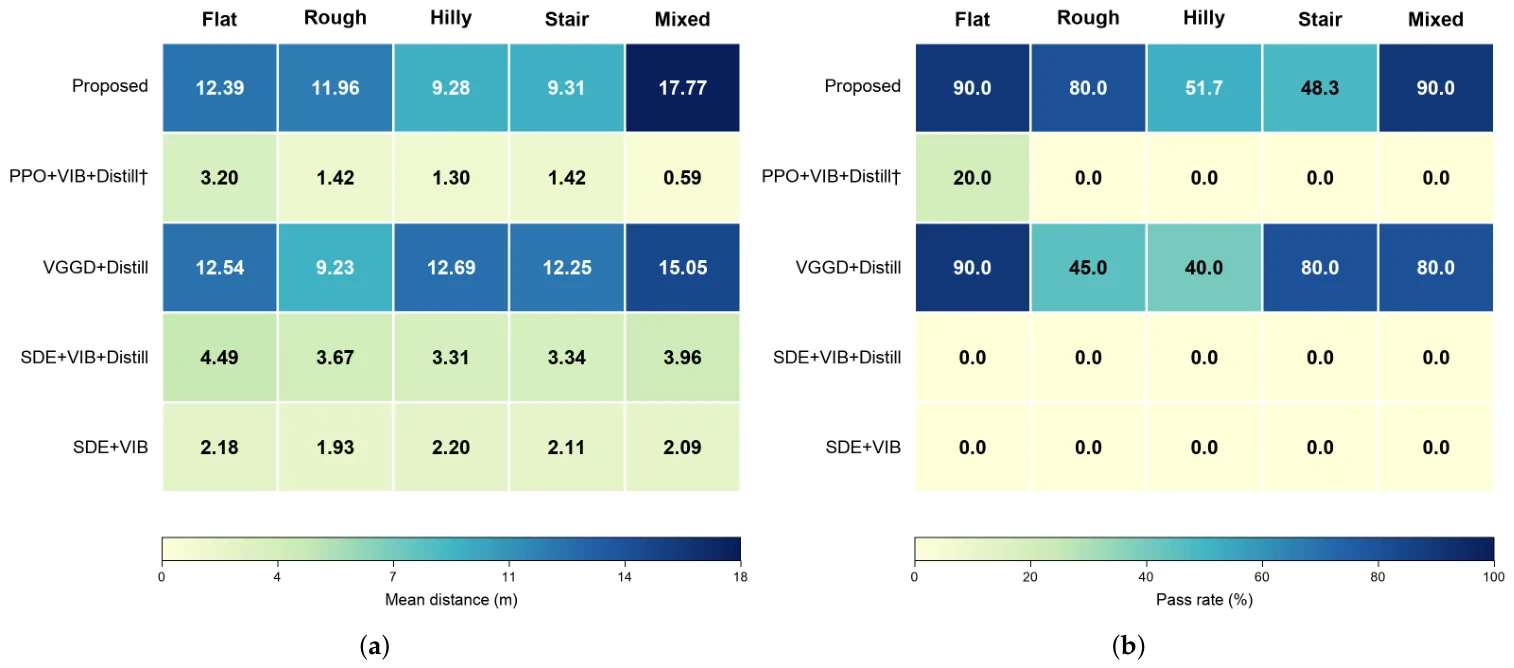
Terrain-wise visualization of the replicated Proposed result (three policies; 60 rollouts per terrain) and the component controls (one policy each; 20 rollouts per terrain). Numerical results for the controls are reported in Table 9. (**a**) Mean distance, (**b**) Pass rate.

Figure 5 presents the archived ablation training curves under the same nominal final-controller budget. The added PPO + VIB + Distill controller achieved 4/100 exact successes (4.0%; rollout-level Wilson 95% CI, 1.6–9.8%) and a 1.58 m mean distance. Its successes occurred on flat terrain (4/20), with no success on rough, hilly, stair, or mixed terrain. The single VGGD + Distill policy achieved 67/100 successes and a 12.35 m mean distance, while the two single-policy SDE controls achieved 0/100 successes. Figure 6 visualizes the replicated Proposed result alongside these controls.

### 5.7. Mixed-Terrain Biomechanical Comparison

The gait plots retain the original selected mixed-terrain traces and are treated as illustrative rather than inferential. In particular, the PPO trace is the originally reported middle-performance rollout (9.58 m), selected near the reported PPO mixed-terrain mean of 9.61 m, rather than a newly substituted maximum-distance rollout. Because the archived traces were not selected by one symmetric rule across all methods, the examples cannot support a method-level biomechanical comparison or cross-method significance test. Method-level conclusions therefore remain based on the task summaries in Table 7. The corresponding original single-rollout descriptors are summarized in Table 10. To quantify whether the Proposed gait descriptors are repeatable beyond one selected trace, Table 11 separately summarizes all retained full gait-variable records from two independently trained Proposed checkpoints and 20 common evaluation seeds per terrain.

#### Multi-Seed Gait Robustness of the Proposed Controller

The archived gait manifests contain complete scalar gait variables for two Proposed checkpoints on the common seed set 1000–1019. We therefore summarize 40 stochastic rollouts per terrain. For each bootstrap replicate, the 20 seed labels are resampled with replacement while the two checkpoint outcomes sharing a seed are kept together; 10,000 resamples yield the bracketed 95% intervals. These intervals quantify evaluation-seed variability averaged over the two retained checkpoints. They are not confidence intervals over a population of trained policies and are not used for cross-method significance claims. The mixed row follows the separate approximately 26 m full-course gait protocol and therefore does not redefine the 13 m main-table pass event.

Across the retained rollouts, stair terrain produces the shortest mean step length, rough terrain produces the largest mean double-support fraction, and hilly and stair terrains produce the largest pelvis-height variation. The full-course mixed rollouts retain similar mean speed and step length but show larger pelvis roll and pitch RMS than flat terrain. These patterns provide multi-seed evidence of terrain-conditioned gait reorganization within the Proposed controller; they do not establish superiority over another method because equivalent population gait manifests are not available for every baseline.

**Figure 7:**
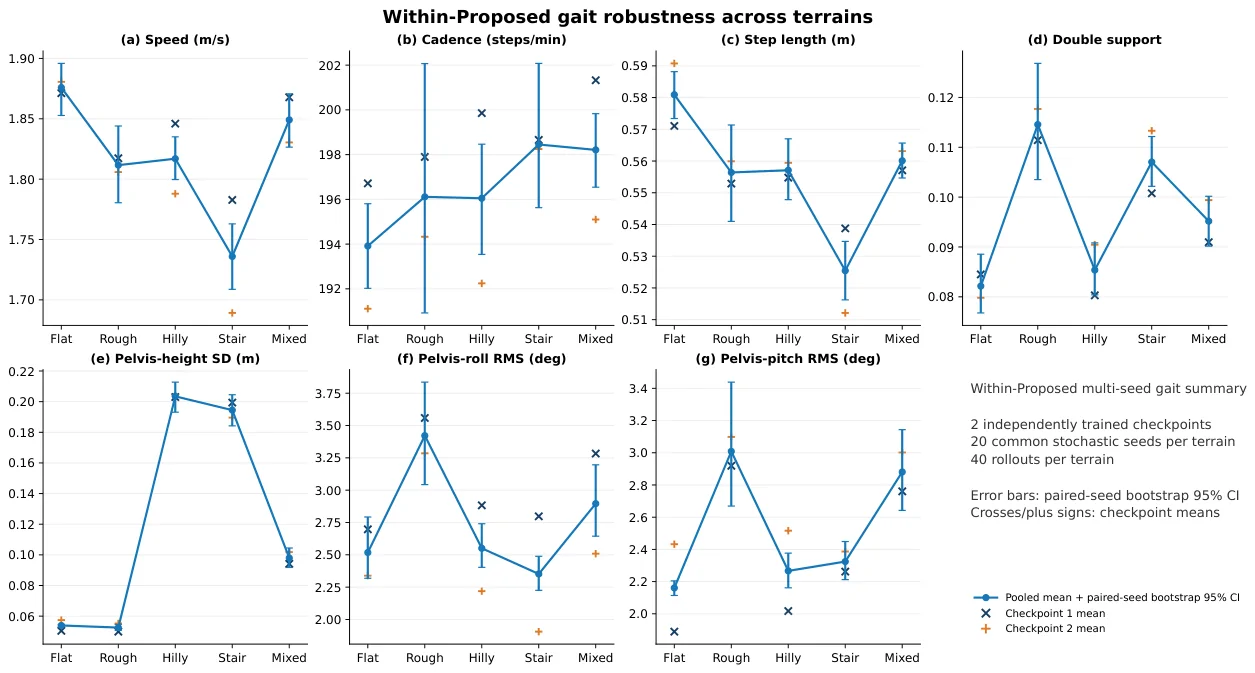
Multi-seed gait robustness of the Proposed controller across five terrains. Points and error bars show the pooled mean and paired-seed bootstrap 95% interval from two independently trained checkpoints evaluated on 20 common stochastic seeds per terrain (40 rollouts per terrain). Cross and plus markers show the two checkpoint-specific means. The figure visualizes the same retained records summarized in Table 11; it quantifies within-Proposed repeatability and is not a cross-method significance comparison.

**Figure 8:**
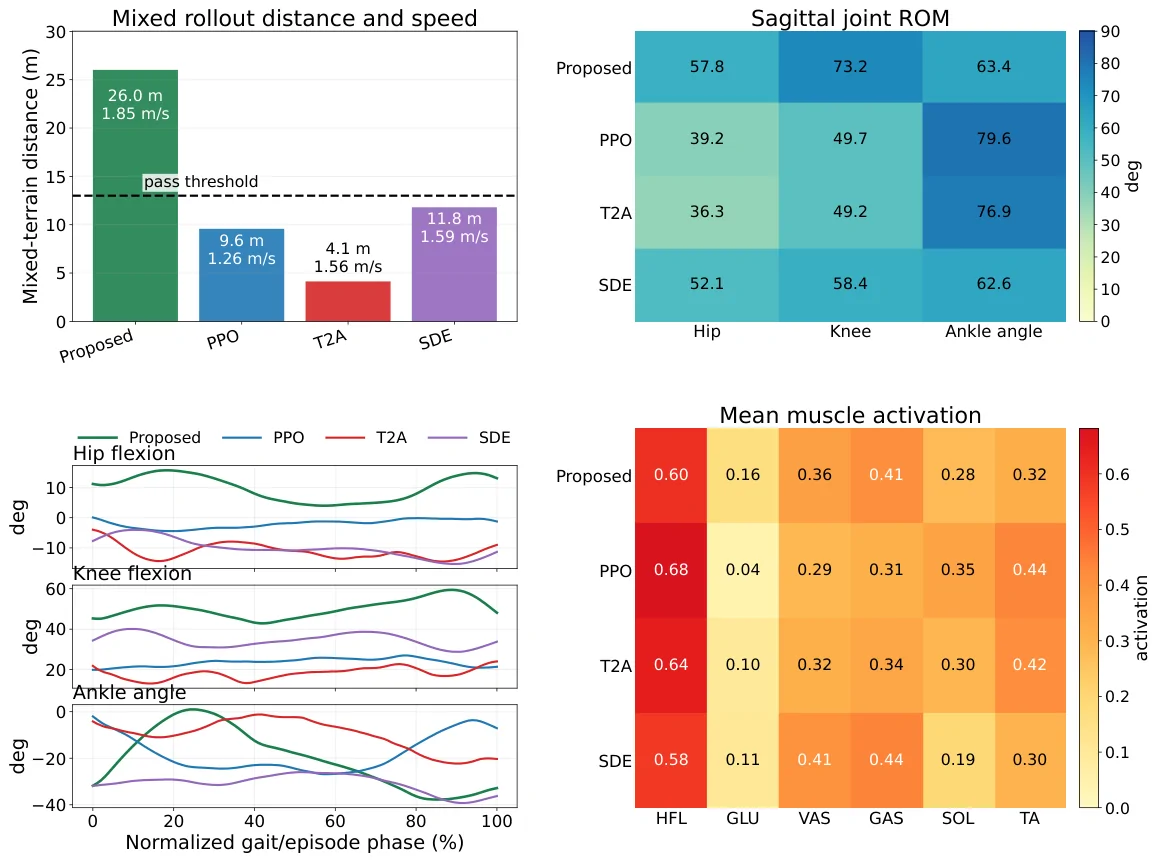
Original selected mixed-terrain traces used for the illustrative biomechanical comparison. PPO is represented by a middle-performance rollout close to its reported mixed-terrain mean. Because the traces do not follow one symmetric population-sampling rule, the panels are not used for population-level inference or method ranking.

For interpretation, speed is the episode-mean forward translational speed; cadence is the detected step count normalized to steps per minute; step length is the mean forward progression per detected step; double support is the fraction of evaluated time in which both feet are in contact; pelvis-height SD measures vertical pelvis excursion; and pelvis roll/pitch RMS measure the root-mean-square lateral tilt and fore–aft tilt, respectively. Lower variability or angular RMS is not universally better: terrain negotiation can require deliberate vertical or rotational motion, so the panels are interpreted jointly with speed, step length, and support timing rather than as seven independent performance scores. Figure 7 visualizes the multi-seed Proposed-controller summaries, and Figure 8 presents the original illustrative cross-method traces.

In the retained examples, Proposed travels 26.02 m with speed 1.85 m/s, step length 0.566 m, and double-support fraction 0.077. The middle-performance PPO rollout travels 9.58 m, close to the reported mixed-terrain mean, with speed 1.26 m/s, step length 0.368 m, and double-support fraction 0.183. T2A and SDE terminate at 4.14 and 11.81 m, respectively; the SDE example also has a pelvis-pitch RMS of 9.21 degrees. These original observations illustrate distinct simulated gait organizations, but differences in episode duration, terrain exposure, and trace-selection criteria preclude inferential biomechanical comparison. The appropriate conclusion is limited to qualitative examples accompanying the task-level summaries, not evidence of human-like gait or statistically superior gait mechanics.

## 6. Discussion

The checkpoint-resolved evaluation files preserve the original aggregate distance means while replacing averaged and rounded pass counts with exact observations. The complete pipeline records 216/300 successful rollouts (72.0%; rollout-level Wilson 95% CI, 66.67–76.78%) and a 12.14 m mean distance, compared with 57/300 (19.0%; 14.96–23.82%) and 6.20 m for nominal-body PPO. Its three independently launched checkpoints contribute 84/100, 78/100, and 54/100 successes, whereas PPO contributes 49/100, 0/100, and 8/100. The exact policy-level tests yield p=0.10 for success and p=0.20 for distance. Among the single-policy controls, VGGD+Distill records 67/100 successes and a 12.35 m mean distance, whereas PPO+VIB+Distill and the evaluated SDE variants record lower outcomes in their retained runs.

Table 3 clarifies the computational accounting of the complete pipeline. Final-controller learning is standardized at 100 M nominal interactions per run, Stage A uses a one-off 10 M interaction budget, and Stage B performs proxy-only optimization without MuJoCo calls. Expert costs are shown both as reusable pretrained artifacts and under full-cost assignment. The archived wall-clock ranges are also reported with their hardware context. This stage-wise accounting makes the controller-training comparison and the additional design computation directly auditable.

The constrained T2A and SDE implementations should not be interpreted as evaluations of the native algorithms under their preferred adaptive settings. We intentionally require one fixed test body to match a deployable musculoskeletal system, thereby removing online morphology switching. This makes the comparison relevant to the paper’s fixed-body question, but may disadvantage morphology-adaptive baselines. Their low pass counts support only the statement that these constrained configurations were unsuccessful under the present protocol.

The independent value-proxy experiment removes the selection bias of the earlier all-candidate diagnostic. Across 100 random designs excluded from calibration and Stage-B optimization, Pearson and Spearman coefficients are consistently positive for aggregate mean return, worst-terrain return, and every primitive terrain; affine calibration fitted on a separate 50-design set yields small mean test-set bias and moderate prediction error. The nominal and selected designs are retained only as additional cases. Under the frozen Stage-A policy, the selected anchor has a higher proxy score and a similar worst-terrain return but does not exceed the nominal anchor in mean return, so these two cases are not used to claim isolated design-stage improvement. The paired equal-distance experiment provides the more direct directional test: at all three anchors, the positive proxy perturbation improves mean and seed-wise worst-terrain return relative to the negative perturbation, with all paired-bootstrap intervals above zero. The 32-design neighborhood further supports local mean-return ranking, while its worst-terrain ranking is less precise. Taken together, the evidence supports the proxy as a statistically validated design-ranking signal and a locally useful search direction, without equating it with the analytical simulator-return gradient or a guarantee of global optimality.

The actuator parameters are bounded simulator multipliers, with nominal value one and explicit ranges in Equation (2). Their association with force capacity, contraction velocity, and passive response is mechanically interpretable, but the bounds are not subject-specific physiological ranges and have not been validated against human parameter distributions. The local direction experiment now provides controlled sensitivity evidence for equal-radius perturbations of the nominal, midpoint, and selected latent vectors. Broader robustness to the proximity coefficient, PCA basis, and channel ranges remains untested and should be evaluated before drawing a physiological-optimality conclusion.

Finally, the mixed-terrain gait panels retain the original selected examples, including the middle-performance PPO trace. Because the cross-method traces were not sampled by one symmetric population rule, they are not used for method ranking. Separately, the retained Proposed-controller gait files support a multi-seed repeatability analysis over two independently trained checkpoints and 20 common evaluation seeds per terrain. Its paired-seed bootstrap intervals indicate terrain-conditioned changes—including shorter steps on stairs, greater double support on rough terrain, and larger pelvis-height variation on hilly and stair terrain—but cannot establish superiority over the baselines. The biomimetic contribution is therefore limited to fixed muscle-driven parameterization and simulation-level functional organization; it is not a claim of human gait reproduction or hardware transfer.

## 7. Conclusions

This paper presented VGGD, a proxy-guided framework for selecting a single fixed muscle–tendon parameterization before cross-terrain controller learning. The method searches a compact PCA manifold using a soft-worst objective, quadratic proximity regularization, projected Adam updates, and the final iterate of a finite search; it does not claim a global maximizer or a true simulator-return gradient.

Independent testing on 100 random designs, with calibration fitted on a disjoint 50-design set, shows moderate and statistically significant agreement between the frozen proxy and mean, worst-terrain, and terrain-specific rollout returns. The paired local direction validation further reduces the concern that frozen-buffer evaluation necessarily produces an incorrect update direction: using equal feasible-space perturbation radii and 60 previously unused paired evaluation seeds, the proxy direction agrees with improvements in mean and seed-wise worst-terrain rollout return at the nominal, midpoint, and selected designs. The complementary 32-design neighborhood supports local mean-return ranking and quantifies the remaining uncertainty for finer worst-terrain ordering.

Across flat, rough, hilly, stair, and mixed terrains, the complete pipeline retains the original 72.0% pooled pass rate and 12.14 m mean distance, now resolved as 216/300 observed successes contributed by checkpoint totals of 84/100, 78/100, and 54/100. Nominal-body PPO retains 19.0% and 6.20 m, resolved as 57/300 successes contributed by 49/100, 0/100, and 8/100. Exact policy-level permutation tests yield p=0.10 for success and p=0.20 for distance. The four single-policy component controls are reported separately with equal within-table replication.

The originally selected cross-method gait traces provide qualitative simulation examples, while the separate Proposed-controller analysis over two checkpoints and 20 common evaluation seeds per terrain adds uncertainty intervals for gait repeatability. Together, the proxy validation and local-direction test support the proposed design-search mechanism, while the task and gait results document the behavior of the fixed-body design–control pipeline across heterogeneous terrain. Future work will extend the design space and evaluate the resulting morphology–control configurations on physical musculoskeletal platforms.

## Figures and Tables

**Table 1:** Nominal and selected latent design values and the resulting range across the 40 bilateral muscle groups. Decoded entries report minimum/mean/maximum scale.

Channel	Selected Latent Coordinates	Nominal Scale	Selected Decoded Scale
Strength $z_{\sigma }$	[−1.0000,−0.7848,−1.0000,−1.0000,−1.0000]	1.000	0.869/0.999/1.283
Velocity $z_{\nu }$	[−0.6589,−1.0000,1.0000,1.0000,0.4187]	1.000	0.780/1.010/1.292
Passive $z_{\kappa }$	[1.0000,−0.6803,1.0000,−1.0000,1.0000]	1.000	0.864/1.012/1.122

**Table 2:** Implementation and evaluation settings.

Item	Setting
Environment	myoLegLongTerrainWalk
Actuators	80 muscle excitations
Design latent	15-dimensional inherited PCA basis: five coordinates each for strength, velocity and passive-force scale channels
Scale ranges	σ∈(0.8,1.5), ν∈(0.5,1.5) and κ∈(0.8,1.2); nominal scale is 1.0 for every channel
Design-stage terrains	flat, rough, hilly and stair
Stage-C and evaluation terrains	the four primitive terrains plus a mixed course that randomly orders primitive sections
Mixed course	approximate total traversal length of 26 m
Observation	proprioceptive state plus local 11×11 terrain height map for terrain-aware policies; no-terrain baselines omit the height-map input
Policy optimizer	PPO with MultiInputPolicy and two 64-unit layers for the policy and value heads, as instantiated in the archived SB3 checkpoints
Training budget	Final controller training: nominally 100 M environment steps per independently launched run with 32 parallel environments; SB3 rollout-block rounding gives 100.008 M archived steps per completed baseline run
Design-stage overhead	Stage-A value-proxy learning uses a 10 M environment-step budget with 8 parallel environments and a three-head value ensemble; Stage B uses a saved cross-terrain buffer and 200 projected Adam updates without MuJoCo calls
PPO settings	learning rate $10^{-4}$, batch size 2048, γ=0.995, GAE λ=0.95
VIB/distillation	KL bottleneck on terrain latent code and optional action-space expert imitation regularizer in Stage C
Evaluation	three independent training runs per main method; 20 stochastic rollout seeds per checkpoint and terrain; exact pooled and checkpoint-specific successes, rollout-level Wilson intervals, between-checkpoint SDs, checkpoint-bootstrap intervals, and exact policy-level permutation tests are reported
Pass count	rollout reaches the predefined 13 m target; the approximately 26 m mixed course is retained as a separate full-course gait protocol
Hardware	archived Stage-C logs: Python 3.9.25; Proposed on an NVIDIA RTX 4070 Laptop GPU host (CUDA 12.2), no-terrain baselines on a four-RTX-3090 host (CUDA 12.4); wall-clock values are therefore hardware-specific

**Table 3:** Training -time environment interactions and non-simulator computation. Values are totals for the reported three-training-run main comparison unless a stage is explicitly one-off.

Method or Stage	Repetitions	Parallel Envs	Environment Interactions (M)	MuJoCo Calls During Optimization	Additional Compute Record
VGGD Stage A	one-off	8	10.000	Yes	confirmed Stage-A budget; three-head value-proxy learning
Cross-terrain state buffer	4×20×5 episodes	1	0.0118	Yes	11,750 stored simulator states
VGGD Stage B	200 updates	–	0	No	204,800 critic-state evaluations
Terrain-expert pretraining	four experts	32	300.024	Yes	100/100/50/50 M nominal steps
Proposed Stage C	three training runs	32	300.024	Yes	11.89–14.10 h recorded over 100.008–118.807 M logged steps; RTX 4070 Laptop GPU
Proposed, reusable-teacher total	–	–	310.036	–	Stage A + buffer + Stage C
Proposed, full-cost total	–	–	610.060	–	includes all expert pretraining
PPO total	three training runs	32	300.024	Yes	30.61–30.96 h; RTX 3090 host; final-controller training only
PPO+VIB+Distill control	one training run	32	100.008	Yes	nominal body; archived controller
T2A total	three training runs	32	300.024	Yes	30.60–31.44 h; RTX 3090 host; final-controller training only
SDE total	three training runs	32	300.024	Yes	30.89–31.12 h; RTX 3090 host; final-controller training only

**Table 4:** Independent value-proxy validation on 100 held-out random designs. Brackets are design-level bootstrap 95% confidence intervals from 10,000 resamples. MAE and RMSE are computed after affine calibration on a separate 50-design set. $p_{\mathrm{adj}}$ is the Holm-adjusted two-sided permutation value across the six reported targets.

Rollout Target	Pearson *r* [95% CI]	Spearman ρ [95% CI]	MAE [95% CI]	RMSE [95% CI]	$R^{2}$	$p_{\mathrm{adj}}$
Mean across terrains	0.580 [0.445, 0.693]	0.565 [0.413, 0.687]	9.00 [7.80, 10.57]	11.19 [9.86, 12.87]	0.336	0.0006
Worst-terrain return	0.583 [0.447, 0.696]	0.551 [0.397, 0.670]	8.11 [7.03, 9.49]	10.00 [8.86, 11.50]	0.340	0.0006
Flat	0.589 [0.461, 0.699]	0.567 [0.424, 0.686]	8.07 [7.01, 9.46]	9.91 [8.76, 11.46]	0.346	0.0006
Rough	0.563 [0.422, 0.683]	0.536 [0.374, 0.668]	10.09 [8.74, 12.02]	12.84 [11.24, 14.98]	0.314	0.0006
Hilly	0.546 [0.397, 0.674]	0.524 [0.364, 0.656]	10.76 [9.32, 12.54]	13.25 [11.70, 15.13]	0.296	0.0006
Stairs	0.576 [0.440, 0.687]	0.544 [0.391, 0.665]	8.42 [7.33, 9.81]	10.35 [9.24, 11.85]	0.330	0.0006

**Table 5:** Paired equal-distance local proxy-gradient validation with 60 previously unused evaluation seeds. Proxy and rollout differences are positive-direction minus negative-direction; the final comparison column is positive-direction minus the unperturbed anchor. Brackets give paired-bootstrap 95% confidence intervals.

Anchor	Proxy Δ	Mean-Return Δ [95% CI]	Worst-Terrain Δ [95% CI]	Positive–Anchor Δ [95% CI]	Mean Δ>0	Worst Δ>0
Nominal	0.02370	1.593 [1.243, 2.016]	1.131 [0.795, 1.509]	0.733 [0.521, 1.024]	60/60	53/60
Midpoint	0.02380	1.169 [0.926, 1.419]	1.162 [0.792, 1.615]	0.623 [0.458, 0.784]	56/60	53/60
Selected	0.01882	1.034 [0.828, 1.248]	1.012 [0.723, 1.328]	0.455 [0.306, 0.623]	57/60	52/60

**Table 6:** Components in the reported main comparison. A check mark indicates that the component is enabled, whereas a dash indicates that it is absent.

Method	VGGD-Selected Body	11×11 Terrain Map	VIB	Distillation	Fixed Test Body
Proposed	✓	✓	✓	✓	✓
PPO	–	–	–	–	✓
T2A (constrained)	–	–	–	–	✓
SDE (constrained)	–	–	–	–	✓

**Table 7:** Main cross-terrain rollout comparison from checkpoint-resolved evaluation records. Each terrain cell retains the original distance mean ± SD and gives the exact pooled successes across three checkpoints and 20 evaluation seeds per checkpoint. The mixed-terrain count uses the original 13 m evaluation target. The Wilson interval describes pooled rollout outcomes and is not a confidence interval across independently trained policies.

Method	Flat	Rough	Hilly	Stair	Mixed	Exact Total	Rate	95% Wilson CI	Mean Distance
Proposed	12.39±2.21 (54/60)	11.96±2.57 (48/60)	9.28±4.31 (31/60)	9.31±4.14 (29/60)	17.77±5.79 (54/60)	216/300	72.0%	[66.7, 76.8]%	12.14
PPO	8.50±4.35 (26/60)	2.34±1.72 (1/60)	5.41±3.84 (11/60)	5.16±3.53 (6/60)	9.61±7.22 (13/60)	57/300	19.0%	[15.0, 23.8]%	6.20
T2A	2.10±0.48 (0/60)	1.35±0.31 (0/60)	2.08±0.50 (0/60)	1.98±0.35 (0/60)	2.14±0.56 (0/60)	0/300	0.0%	[0.0, 1.3]%	1.93
SDE	5.51±3.80 (5/60)	2.46±1.44 (0/60)	3.43±1.50 (0/60)	3.40±1.86 (0/60)	5.05±2.93 (0/60)	5/300	1.7%	[0.7, 3.8]%	3.97

**Table 8:** Checkpoint -level uncertainty and exact comparisons. Success entries list the three observed checkpoint totals out of 100 rollouts. Bracketed intervals are 10,000-resample checkpoint-bootstrap intervals for the checkpoint mean. Exact *p*-values compare the three Proposed policy summaries with the corresponding three baseline summaries. Because only 63=20 allocations exist, the smallest attainable nonzero two-sided exact *p*-value is 0.10.

Method	Checkpoint Successes (/100)	Policy Pass Rate, Mean ± SD [95% CI]	Policy Mean Distance, Mean ± SD [95% CI]	$p_{\mathrm{pass}}$	$p_{\mathrm{distance}}$
Proposed	84; 78; 54	72.0±15.9 [54.0, 84.0]%	12.14±2.59 [9.15, 13.64] m	–	–
PPO	49; 0; 8	19.0±26.3 [0.0, 49.0]%	6.20±3.36 [3.51, 9.97] m	0.100	0.200
T2A	0; 0; 0	0.0±0.0 [0.0, 0.0]%	1.93±0.29 [1.60, 2.14] m	0.100	0.100
SDE	0; 5; 0	1.7±2.9 [0.0, 5.0]%	3.97±1.83 [1.86, 5.19] m	0.100	0.100

**Table 9:** Single -policy component controls using the available reproducible records. Every row represents one independently trained policy evaluated with 20 stochastic seeds per terrain (100 rollouts total). Cells report mean ± SD and exact success counts. The separately replicated Proposed result is reported in Table 7 and Table 8.

Method	Flat	Rough	Hilly	Stair	Mixed	Total	Rate	95% Wilson Interval	Mean Distance
PPO + VIB + Distill	3.20±5.03 (4/20)	1.42±1.89 (0/20)	1.30±1.02 (0/20)	1.42±1.47 (0/20)	0.59±0.14 (0/20)	4/100	4%	[1.6, 9.8]%	1.58
VGGD + Distill	12.54±1.42 (18/20)	9.23±4.01 (9/20)	12.69±0.34 (8/20)	12.25±1.66 (16/20)	15.05±3.26 (16/20)	67/100	67%	[57.3, 75.4]%	12.35
SDE + VIB + Distill	4.49±1.53 (0/20)	3.67±1.35 (0/20)	3.31±0.58 (0/20)	3.34±0.66 (0/20)	3.96±0.95 (0/20)	0/100	0%	[0.0, 3.7]%	3.75
SDE + VIB	2.18±0.21 (0/20)	1.93±0.30 (0/20)	2.20±0.19 (0/20)	2.11±0.18 (0/20)	2.09±0.29 (0/20)	0/100	0%	[0.0, 3.7]%	2.10

**Table 10:** Spatiotemporal and posture metrics from the original selected mixed-terrain rollout for each method. The PPO trace is a middle-performance rollout near its reported mean. These single-rollout summaries are illustrative and are not estimates of population means.

Method	Distance	Speed	Cadence	Step Length	Double Support	No Contact	Pelvis-Height SD	Roll/Pitch RMS
Proposed	26.02	1.85	196.4	0.566	0.077	0.190	0.104	2.96/2.01
PPO	9.58	1.26	205.8	0.368	0.183	0.136	0.044	2.10/2.35
T2A	4.14	1.56	180.5	0.517	0.247	0.109	0.058	3.32/4.95
SDE	11.81	1.59	226.4	0.422	0.253	0.167	0.063	2.67/9.21

**Table 11:** Proposed -controller gait variables over two independently trained checkpoints and 20 common evaluation seeds per terrain (40 rollouts per terrain). Entries are mean [paired-seed bootstrap 95% CI].

Terrain	Speed (m/s)	Cadence (steps/min)	Step Length (m)	Double Support	Pelvis-Height SD (m)	Roll RMS (deg)	Pitch RMS (deg)
Flat	1.876 [1.853, 1.896]	193.9 [192.0, 195.8]	0.581 [0.573, 0.588]	0.082 [0.077, 0.089]	0.054 [0.053, 0.055]	2.52 [2.32, 2.79]	2.16 [2.11, 2.20]
Rough	1.812 [1.781, 1.844]	196.1 [190.9, 202.1]	0.556 [0.541, 0.571]	0.115 [0.104, 0.127]	0.053 [0.051, 0.055]	3.42 [3.04, 3.84]	3.01 [2.67, 3.44]
Hilly	1.817 [1.800, 1.835]	196.1 [193.5, 198.5]	0.557 [0.548, 0.567]	0.085 [0.080, 0.091]	0.204 [0.193, 0.213]	2.55 [2.40, 2.74]	2.27 [2.16, 2.38]
Stair	1.736 [1.709, 1.763]	198.5 [195.6, 202.1]	0.526 [0.516, 0.535]	0.107 [0.102, 0.112]	0.195 [0.184, 0.205]	2.35 [2.23, 2.49]	2.32 [2.21, 2.45]
Mixed	1.849 [1.827, 1.871]	198.2 [196.5, 199.8]	0.560 [0.555, 0.566]	0.095 [0.090, 0.100]	0.098 [0.092, 0.105]	2.90 [2.64, 3.20]	2.88 [2.64, 3.14]

## Data Availability

The checkpoint-resolved evaluation CSV files used to obtain the exact success counts, policy-level uncertainty summaries, terrain-specific results, and Proposed-controller multi-seed gait intervals are provided with the reproducible analysis script as supplementary material. The independent value-proxy package contains the frozen protocol, 50 calibration designs, 100 held-out test designs, 32 local candidates, 3680 raw rollout records, terrain- and design-level summaries, calibration bins, statistical output, and Supplementary Figures S1 and S2. The paired local-gradient validation records and script are also included. The PPO + VIB + Distill stochastic rollout manifest, its evaluation script, tabulated summary, and archived training diagnostic are supplied. The full training code and checkpoints are available from the corresponding author upon reasonable request and are planned for public release subject to institutional approval.

## Supplementary Materials

The following supporting information can be downloaded at https://www.mdpi.com/article/10.3390/biomimetics11090623/s1, Figure S1: Held-out value-proxy predictions versus rollout returns; Figure S2: affine calibration of the held-out value proxy; Video S1: illustrative cross-terrain rollout of the proposed method; supplementary data and analysis code for the checkpoint-resolved main evaluation, independent value-proxy validation, local gradient-direction validation, multi-seed gait evaluation, and PPO + VIB + Distill evaluation.

## Abbreviations

The following abbreviations are used in this manuscript:

VGGD	Value-Gradient Generalist Design
SDE	Spectral Design Evolution
MTU	Muscle–Tendon Unit
PCA	Principal Component Analysis
PPO	Proximal Policy Optimization
VIB	Variational Information Bottleneck
T2A	Transform2Act
