# Supplementary material for: Value-Gradient Generalist Design of Muscle–Tendon Parameters for Cross-Terrain Musculoskeletal Locomotion
Source: Biomimetics (Basel). 2026 Sep 2;11(9):623. doi: 10.3390/biomimetics11090623 (PMC13604786; doi:10.3390/biomimetics11090623)
Supplement: Supplementary file 1 [file biomimetics-11-00623-s001.zip › Supplementary_Data_and_Code/value_proxy_validation/Supplementary_Figure_S1_Proxy_Scatter.pdf]

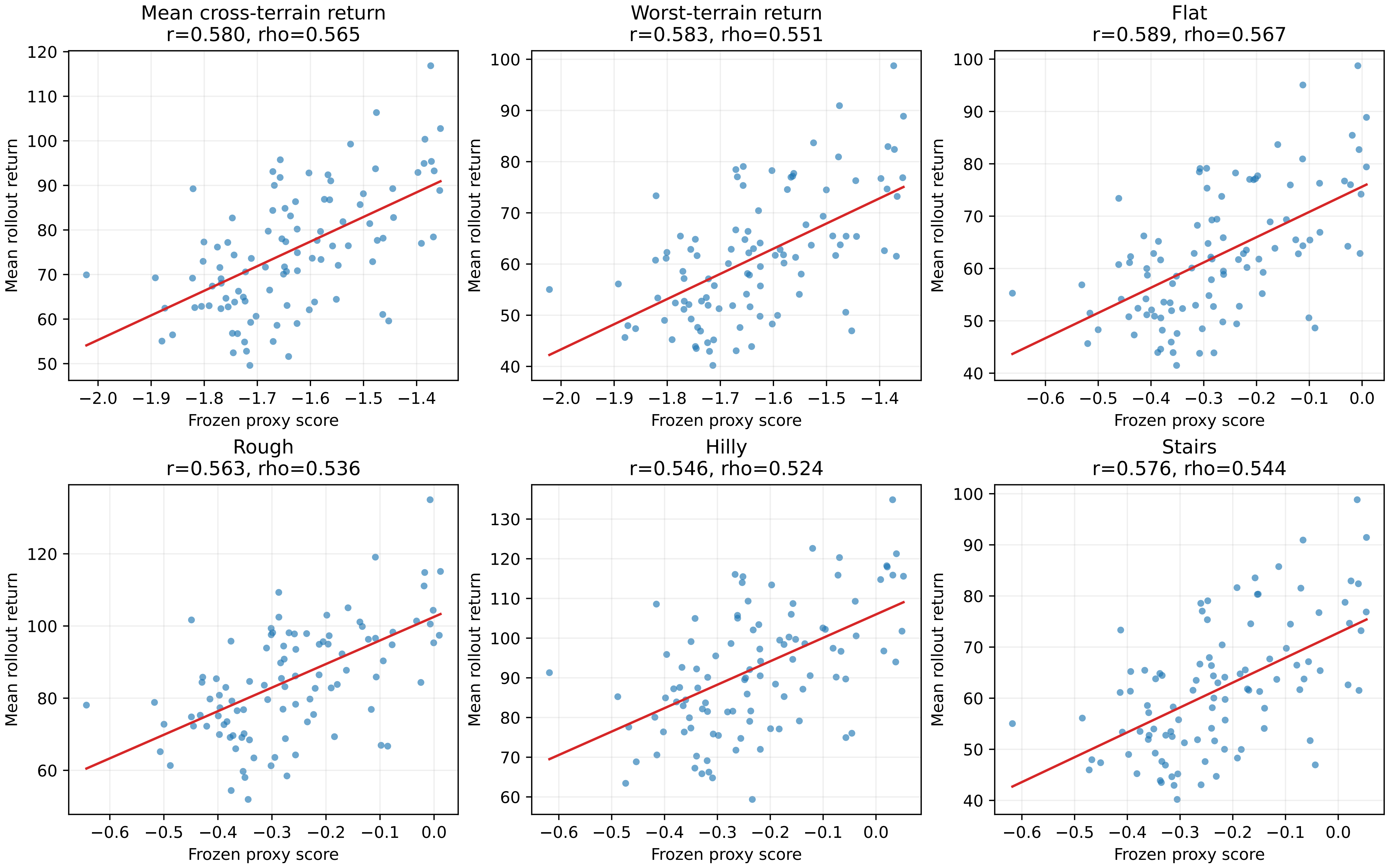
Mean cross-terrain return
r=0.580, rho=0.565
Worst-terrain return
r=0.583, rho=0.551
Flat
r=0.589, rho=0.567
Rough
r=0.563, rho=0.536
Hilly
r=0.546, rho=0.524
Stairs
r=0.576, rho=0.544
Mean rollout return
Frozen proxy score
