# Supplementary material for: Value-Gradient Generalist Design of Muscle–Tendon Parameters for Cross-Terrain Musculoskeletal Locomotion
Source: Biomimetics (Basel). 2026 Sep 2;11(9):623. doi: 10.3390/biomimetics11090623 (PMC13604786; doi:10.3390/biomimetics11090623)
Supplement: Supplementary file 1 [file biomimetics-11-00623-s001.zip › Supplementary_Data_and_Code/value_proxy_validation/Supplementary_Figure_S2_Calibration.pdf]

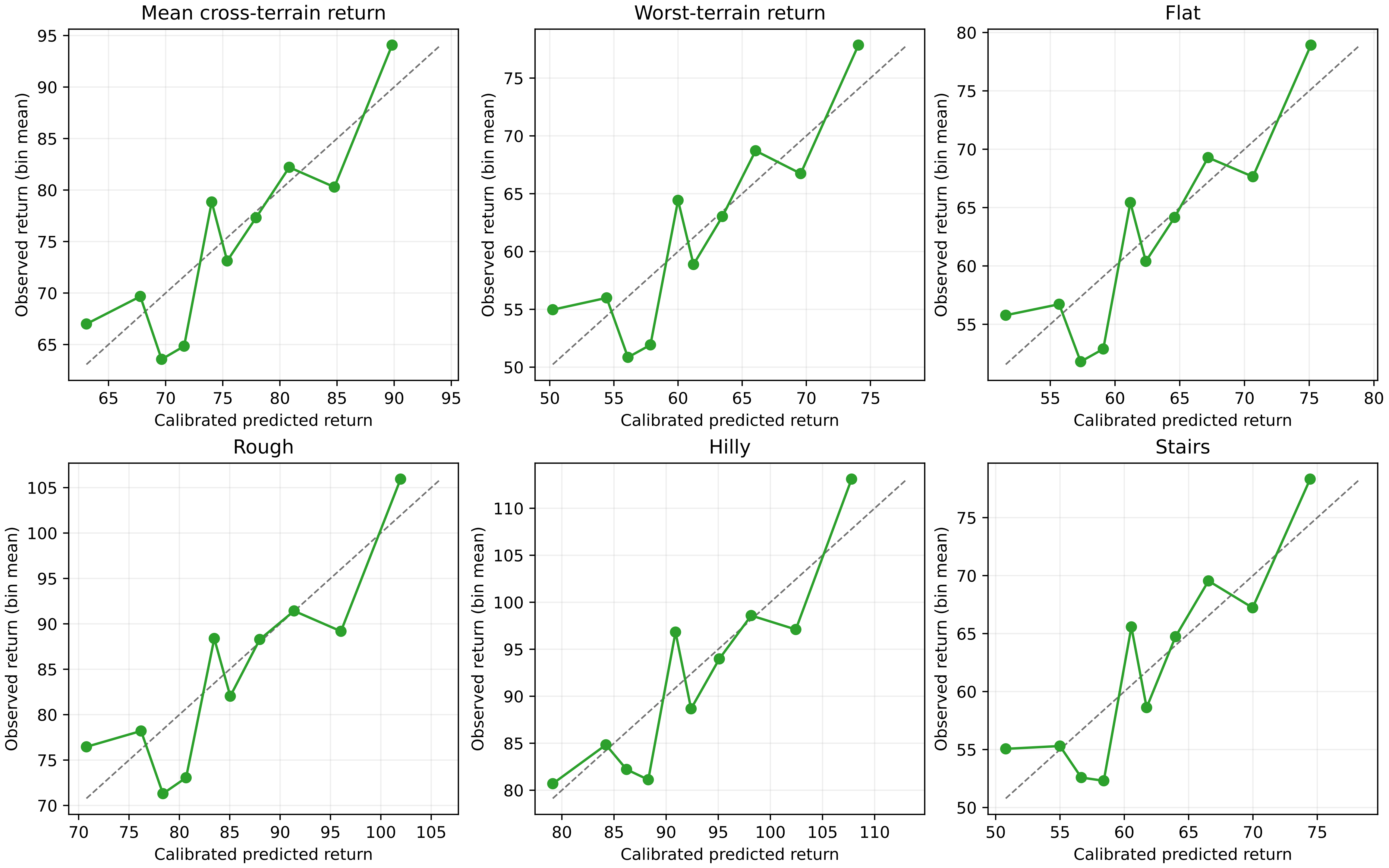

Mean cross-terrain return
Worst-terrain return
Flat
Rough
Hilly
Stairs
Observed return (bin mean)
Calibrated predicted return
